# Clinicopathologic significance and prognostic value of circRNAs in osteosarcoma: a systematic review and meta-analysis

**DOI:** 10.1186/s13018-021-02568-2

**Published:** 2021-10-07

**Authors:** Jingyu Zhong, Guangcheng Zhang, Weiwu Yao

**Affiliations:** 1 Department of Imaging, Tongren Hospital, Shanghai Jiao Tong University School of Medicine, No. 1111 Xianxia Road, Shanghai, 200336 China; 2 Department of Orthopedics, Shanghai Jiao Tong University Affiliated Sixth People’s Hospital, Shanghai, 200233 China

**Keywords:** Osteosarcoma, Circular RNA, Clinicopathology, Overall survival, Disease-free survival, Systematic review, Meta-analysis

## Abstract

**Abstract:**

**Background:**

Osteosarcoma is the most prevalent malignant osseous sarcoma in children and adolescents, whose prognosis is still relatively poor nowadays. Recent studies have shown the critical function and potential clinical applications of circular RNAs (circRNAs) in osteosarcoma. Our review aimed to perform an updated meta-analysis to explore their clinicopathologic significance and prognostic value.

**Methods:**

The structured literature was conducted via eight electronic databases and four gray literature sources until 20 Feb 2021 to identify eligible studies. The data was extracted directly from the articles or reconstructed based on Kaplan-Meier curves. The Newcastle-Ottawa Scale (NOS) tool was used to assess study quality. The clinicopathologic significance of circRNAs was measured through odds ratios (ORs) and their 95% confidence intervals (CIs), while the prognostic value was evaluated through hazard ratios (HRs) and their 95% CIs of overall survival (OS) and disease-free survival (DFS). Heterogeneity and publication bias were assessed. Sensitivity analyses were conducted. Subgroup analyses were performed according to study characteristics. An additional analysis was performed to investigate the relation between circ_0002052 and osteosarcoma.

**Results:**

Fifty-two studies were identified, in which 38 on clinicopathologic features and 36 on survival prognosis were included in quantitative analysis. The overall study quality was moderate with a median NOS score of 5.5 stars (range 3 to 8). For clinicopathologic features, dysregulated circRNAs were related to larger tumor size (OR 2.122, 95%CI 1.418–3.175), advanced clinical stage (OR 2.847, 95%CI 2.059–3.935), and present of metastasis (OR 2.630, 95%CI 1.583–4.371). For chemotherapy, dysregulated circRNAs suggest a better response (OR 0.443, 95%CI 0.231–0.849), but a higher probability of resistance (OR 9.343, 95%CI 5.352–16.309). For survival prognosis, dysregulated circRNAs were significantly correlated with poor OS (HR 2.437, 95%CI 2.224–2.670) and DFS (HR 2.125, 95%CI 1.621–2.786). The results did not show differences among subgroups. Higher circ_0002052 expression showed a relation with poor OS (HR 3.197, 95%CI 2.054–4.976).

**Conclusions:**

Our review demonstrated that abnormally expressed circRNAs have a relation with advanced clinicopathologic features and better response, but a higher probability of resistance and poor survival prognosis in osteosarcoma patients. However, more studies are encouraged to provide more robust evidence to translate circRNAs into clinical practice.

**Trial registration:**

PROSPERO ID: CRD42021235031

**Supplementary Information:**

The online version contains supplementary material available at 10.1186/s13018-021-02568-2.

## Background

Osteosarcoma is a malignant bone tumor characterized by neoplastic bone formation directly from tumor cells [1], which presents the most common primary osseous sarcoma in children and adolescents [2]. The diagnostic work-up of osteosarcoma usually started with radiological examinations for detecting the local diseases, followed by checkup for distant metastases, and finalized with a biopsy to reach a histology diagnosis [2–4]. Although this approach can guide the clinician to an appropriate treatment plan, the 5-year survival rate is still unsatisfying and the etiology of osteosarcoma remains unclear [1, 5]. Current clinicopathologic features and regular tests show potentials in patient prognosis prediction [6], but are unable to reveal the pathogenesis of osteosarcoma. Therefore, it is urgent to identify new biomarkers related to prognosis and clinicopathologic features.

With the development of sequencing technologies, several non-coding RNAs were discovered. Non-coding RNAs participate and regulate the transcription and translation of genes and sometimes play significant roles during dysregulated gene expression in cancer [7, 8]. Circular RNA (circRNA) is one of the non-coding RNAs with a closed loop that is generated by the back-splicing of pre-RNA with covalent bonding in between, functions as a sponge for microRNA, or directly regulates transcription and interfering with splicing mechanisms [9]. Studies have shown that circRNA can serve as diagnostic, prognostic, and predictive biomarkers [10–12]. Further, circRNA may be a more detectable biomarker for cancer, since it has the characteristics of a stable structure that is resistant to degradation by most RNA decay machinery [13–15].

The relation between circRNAs and osteosarcoma has been present in several reviews [16–20]. CircRNAs play oncogenic roles or show tumor-suppressive effects in the pathogenesis and progression of osteosarcoma including cell apoptosis, invasion, growth, differentiation, and migration. They are also involved in malignant phenotypes of osteosarcoma, such as treatment resistance and metastasis. Further quantitative analysis showed the potential of circRNAs in clinical implication as diagnostic or prognostic biomarkers [21, 22]. However, the previous meta-analyses included a number of studies that did not analyze the relation between circRNAs and treatment response and failed to pool repeatably studied circRNAs. Therefore, our systematic review and meta-analysis aimed to provide a more up-to-date and comprehensive summary of the clinicopathologic significance and prognostic value of circRNAs in osteosarcoma.

## Methods

### Protocol and registry

The reporting of our review followed Preferred Reporting Items for Systematic Reviews and Meta-analysis (PRISMA) statement and several extensions [23]. A checklist was presented as Additional file 1. A protocol has been drafted before our review started and has been registered and updated on the International Prospective Register of Systematic Reviews (PROSPERO) [24] as CRD42021235031.

### Literature search

Our systematic literature search was performed by two independent reviewers until 20 Feb 2021 following the Peer Review of Electronic Search Strategies (PRESS) guideline [25]. We searched eight electronic databases including PubMed, Embase, The Cochrane Library, Web of Science, Scopus, SinoMed, China National Knowledge Infrastructure (CNKI), and WanFang databases, as well as four gray literature sources namely OpenGrey, British Library Inside, ProQuest Dissertations & Theses Global, and BIOSIS preview. A search string was firstly developed in PubMed using two key terms, namely circular RNA and osteosarcoma in free words, Medical Subject Headings (MeSH) and/or Emtree words. The search string used in PubMed was (“RNA, Circular”[Mesh] OR circRNA OR ciRNA OR (circular AND RNA) OR “circular ribonucleic acid”) AND (“osteosarcoma”[Mesh] OR osteosarcoma OR (osseous AND sarcoma) OR (osteogenic AND sarcoma)). Then, the search strings were modified into other data sources (Additional file 1). There was no limitation for the time period, study design, or languages during the literature search. Duplicates were excluded through a rigorous and reproducible method via Endnote software version X9.2 (Clarivate Analytics, Philadelphia, PA, USA) [26].

### Study selection

Two reviewers separately screened the titles and abstracts of records from electronic databases after deduplication. The records from gray literature sources were directly screened online to identify additional relevant records. The full texts and supplementary materials of potentially eligible records were obtained by two same reviewers and further assessed for eligibility. The reference lists of included studies and relevant reviews were screened to identify additional eligible studies. In the case of uncertainties, a final consensus was reached through discussion or help from a third reviewer.

Our study inclusion criteria included (1) study with histologically diagnosed osteosarcoma patients; (2) circRNA expression detected using tissues, serum, or plasma; (3) analysis about circRNA on clinicopathologic features or survival prognosis performed. Our study exclusion criteria were (1) ex vivo study or animal study; (2) duplicate studies; (3) reviews, conference abstracts, book chapters, editorials, letters, case reports, and other unsuitable article types; (4) reported in a language other than English, Japanese, Chinese, German, or French.

### Data extraction

Data extraction was independently completed by two reviewers with our standardized sheet. The data extraction sheet contains the following items: (1) bibliographic data: author, publication year, study country; (2) circRNA characteristics: circRNA type, regulation pattern, sample size, specimen type, detection method, cutoff value, number of patients with high or low circRNA expression; (3) clinicopathologic data: age, gender, tumor site, tumor size, clinical stage, histologic classification, differentiation, metastasis; and (4) prognostic information: overall survival (OS), disease-free survival (DFS) or progression-free survival (PFS), hazard ratio (HR) and its 95% confidence interval (CI) for prognostic outcome, analysis method, data availability, follow-up duration. Any disagreement was resolved by discussion or help from a third reviewer.

If the studies have reported prognostic information in the article, we documented the data directly; otherwise, we extracted available data from the Kaplan-Meier curve (K-M curve) via an open-source Engauge Digitizer software version 12.1 [27]. The Engauge Digitizer digitizes image files containing graphs by placing points along axes and curves and recovers the data points from those graphs. Then, we reconstructed the necessary data through several established practical methods for meta-analysis [28] (Supplementary Note 2). The corresponding authors were contacted to request the data, if the articles did not report sufficient data or impossible to reconstruct based on reported data. When there was no response, the article was only qualitatively analyzed.

### Quality assessment

Two reviewers independently assessed the quality of included studies conducting the Newcastle-Ottawa Quality Assessment Scale (NOS) [29, 30]. NOS used a star system to judge the study on three broad perspectives: the selection of the study groups; the comparability of the groups; and the ascertainment of either the exposure or outcome of interest for case-control or cohort studies, respectively. In our review, studies with prognostic outcomes were treated as cohort studies, while those only reported cross-sectional clinicopathologic features were considered as case-control studies. A modified version of NOS was used in our review (Supplementary Table 1). If there were disagreements between the two reviewers, they would be resolved through discussion or consultation with a third reviewer.

### Data synthesis and analysis

The meta-analysis was conducted with Stata software version 15.1 (Stata Corp., College Station, TX, USA) using relevant packages (Supplementary Note 3). A p value < 0.05 suggested statistical significance, unless otherwise specified. To merge the outcomes of up- and downregulated circRNAs, we translated the HRs and 95%CI into a form that HRs > 1 suggested poor prognosis and was considered statistically significant if the 95%CI did not contain 1. The heterogeneity was assessed through the Higgins I-square statistic and chi-square Q test. A random-effect model was applied with the existence of marked heterogeneity as I-square > 50% and chi-square Q p value < 0.10; otherwise, a fixed-effect model was used. The publication bias was objectively evaluated by funnel plots and Begg’s funnel plots. Begg’s and Egger’s tests were quantitatively conducted to detect underlying publication bias. A p value > 0.1 was considered as low publication bias. By omitting the included studies one by one, the reliability of the pooled effect size was assessed. A trim and fill method was also used to assess the reliability of results. Subgroup analyses were performed to explore potential sources of heterogeneity, according to (1) regulation pattern: upregulated, or downregulated; (2) sample size: < 53 samples (median), or ≥ 53 samples; (3) data availability: reported or K-M curve; (4) cutoff value: median, average, or others; and (5) NOS: score < 5.5 stars (median), score ≥ 5.5 stars. An additional analysis was performed to investigate the relation between circ_0002052 and osteosarcoma, since the data from multiple studies allowed a more convictive conclusion.

## Results

### Literature search

**Fig. 1:**
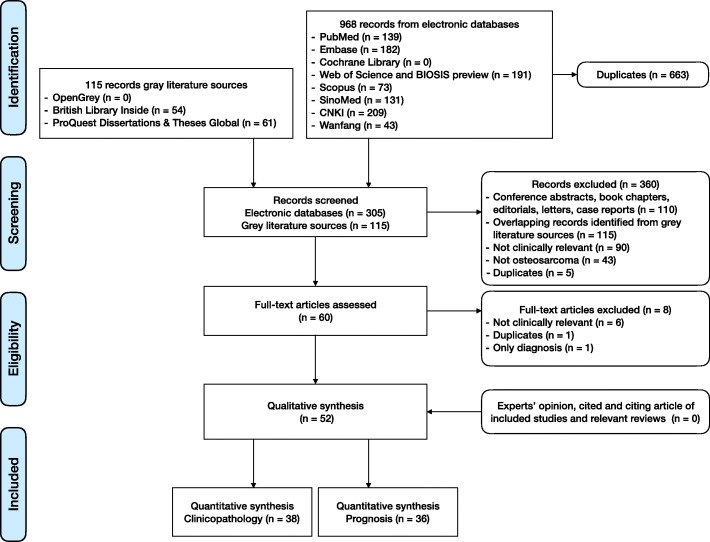
The flow diagram of studies inclusion

As the flow diagram shows (Fig. 1), our systematic review identified 968 records from electronic databases. We screened 305 titles and abstracts after the exclusion of 663 duplicates. Sixty articles were considered to be potentially eligible. We further identified 115 records from gray literature sources; however, no additional eligible article was found. Full-text assessment included 60 articles, and hand search did not identify additional relevant articles. Finally, 52 articles were included in the qualitative analysis [31–82]. Thirty-eight articles on clinicopathology and 36 articles on prognosis were included in the quantitative analysis after the exclusion of articles with incomplete data.

### Study characteristics

Table 1 summarizes the characteristics of included studies. Fifty-two studies with 2934 osteosarcoma patients were included. All the studies were conducted in China. Forty-eight and 4 articles were published in English and Chinese, respectively. Forty-three dysregulated circRNAs were detected, in which 7 were downregulated and 36 were upregulated in osteosarcoma patients. Fifty-one studies measured circRNA expression in tissue samples from osteosarcoma patients by qRT-PCR, while one study used serum as a test sample.

**Table 1:** Characteristics of included studies

Author	Year	CircRNA	Regulation pattern	Country	Sample size	Specimen	Method	Outcome	NOS
Chen	2021	circ_0000885	Upregulated	China	30	Tissue	qRT-PCR	CP	5
Ding	2020	circ_0005909	Upregulated	China	54	Tissue	qRT-PCR	CP, OS	5
Gao	2020	circ_0001721	Upregulated	China	56	Tissue	qRT-PCR	CP, OS	4
Hu	2020	circLARP4	Downregulated	China	72	Tissue	qRT-PCR	CP, DFS, OS	6
Huang	2018	circNASP	Upregulated	China	39	Tissue	qRT-PCR	CP	6
Ji	2020	circ_001621	Upregulated	China	30	Tissue	qRT-PCR	CP, OS	6
Jiang	2020	circXPO1	Upregulated	China	52	Tissue	qRT-PCR	DFS, OS	5
Jiang	2021	circ_0000658	Downregulated	China	60	Tissue	qRT-PCR	CP, OS	4
Jin	2019A	circ_0102049	Upregulated	China	76	Tissue	qRT-PCR	CP, OS	5
Jin	2019B	circ_100876	Upregulated	China	48	Tissue	qRT-PCR	CP, OS	5
Jin	2019C	circ_0002052	Downregulated	China	46	Tissue	qRT-PCR	CP, OS	6
Lei	2020	circ_0003074	Upregulated	China	60	Tissue	qRT-PCR	CP, DFS, OS	6
Li	2018	circ_0007534	Upregulated	China	57	Tissue	qRT-PCR	CP, OS	6
Li	2019	circ_0001721	Upregulated	China	52	Tissue	qRT-PCR	CP, OS	6
Li	2020A	circ_0000073	Upregulated	China	25	Tissue	qRT-PCR	OS	5
Li	2020B	circ 0003732	Upregulated	China	46	Tissue	qRT-PCR	CP, OS	4
Li	2020C	circ_0000190	Downregulated	China	60	Tissue	qRT-PCR	CP	6
Liu	2020	circ_100284	Upregulated	China	52	Tissue	qRT-PCR	CP, OS	4
Liu	2021A	circ_0105346	Upregulated	China	40	Tissue	qRT-PCR	CP, OS	6
Liu	2021B	circMTO1	Downregulated	China	70	Tissue	qRT-PCR	CP, OS	5
Ma	2018	circHIPK3	Downregulated	China	82	Tissue	qRT-PCR	CP, OS	6
Mao	2021	circXPR1	Upregulated	China	20	Tissue	qRT-PCR	DFS, OS	5
Nie	2018	circNT5C2	Upregulated	China	170	Tissue	qRT-PCR	CP, DFS, OS	7
Pan	2019	circMMP9	Upregulated	China	51	Tissue	qRT-PCR	CP, OS	4
Pan	2020	circ_103801	Upregulated	China	43	Serum	qRT-PCR	CP, OS	3
Qi	2018	circ_0000502	Upregulated	China	63	Tissue	qRT-PCR	CP, OS	6
Wang	2019A	circ_0003998	Upregulated	China	60	Tissue	qRT-PCR	OS	5
Wang	2019B	circ_0002052	Downregulated	China	60	Tissue	qRT-PCR	CP, OS	7
Wang	2019C	circ_0021347	Downregulated	China	35	Tissue	qRT-PCR	OS	3
Wang	2020A	circCNST	Upregulated	China	126	Tissue	qRT-PCR	CP, OS	6
Wang	2020B	circTCF25	Upregulated	China	50	Tissue	qRT-PCR	CP	6
Wang	2020C	circ_0001658	Upregulated	China	39	Tissue	qRT-PCR	CP	6
Wei	2021	circ_0081001	Upregulated	China	63	Tissue	qRT-PCR	OS	5
Wen	2021	circHIPK3	Upregulated	China	12	Tissue	qRT-PCR	OS	3
Wu	2020	circ_0002052	Downregulated	China	54	Tissue	qRT-PCR	PFS, OS	3
Xiang	2020	circ_0005721	Upregulated	China	50	Tissue	qRT-PCR	CP, DFS, OS	8
Yan	2020	circPVT1	Upregulated	China	48	Tissue	qRT-PCR	CP, OS	4
Yang	2020	circ_0001105	Upregulated	China	120	Tissue	qRT-PCR	CP, DFS, OS	5
Zhang	2017	circUBAP2	Upregulated	China	92	Tissue	qRT-PCR	OS	4
Zhang	2018	circ_001569	Upregulated	China	36	Tissue	qRT-PCR	CP	8
Zhang	2019	circ_0051079	Upregulated	China	105	Tissue	qRT-PCR	OS	4
Zhang	2020A	circ_0002052	Upregulated	China	40	Tissue	qRT-PCR	CP, OS	4
Zhang	2020B	circ_0136666	Upregulated	China	47	Tissue	qRT-PCR	OS	3
Zhang	2020C	circ_0017247	Upregulated	China	46	Tissue	qRT-PCR	CP	7
Zhang	2021	circ_0005909	Upregulated	China	30	Tissue	qRT-PCR	CP	7
Zhao	2019	circSAMD4A	Upregulated	China	NR	Tissue	qRT-PCR	OS	3
Zheng	2019	circLRP6	Upregulated	China	50	Tissue	qRT-PCR	DFS, OS	4
Zhou	2017	circ_0008717	Upregulated	China	45	Tissue	qRT-PCR	PFS, OS	6
Zhu	2018A	circPVT1	Upregulated	China	80	Tissue	qRT-PCR	CP, OS	6
Zhu	2018B	circ_0081001	Upregulated	China	82	Tissue	qRT-PCR	CP, OS	7
Zhu	2018C	circ_0004674	Upregulated	China	60	Tissue	qRT-PCR	CP, OS	6
Zhu	2019	circ_0000885	Upregulated	China	50	Tissue	qRT-PCR	CP, DFS, OS	6

*CP* clinicopathology, *DFS* disease-free survival, *NA* not applicable, *NOS* Newcastle-Ottawa Scale, *NR* not reported, *OS* overall survival, *PFS* progression-free survival, *qRT-PCR* quantitative real-time polymerase chain reaction

### Quality assessment

**Fig. 2:**
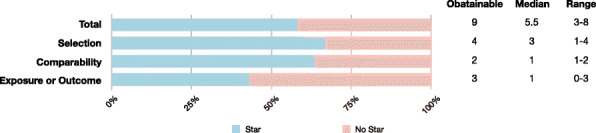
Quality assessment and inter-reviewer agreement of included studies according to the Newcastle-Ottawa Scale

The sum of the NOS score is present in Table 1 and Fig. 2. The sum of the NOS score ranged from 3 to 8 stars, with a median of 5.5 stars, indicating the moderate quality of selected studies. The risk of bias was found mainly related to unclear patient inclusion criteria, inadequate treatment procedure, unreported cutoff value of circRNAs, and various cutoff values of clinicopathologic features, as well as unclear follow-up plan and high loss rate. Detailed quality assessment results are presented in Supplementary Table 2.

### CircRNAs and clinicopathologic features of osteosarcoma

**Fig. 3:**
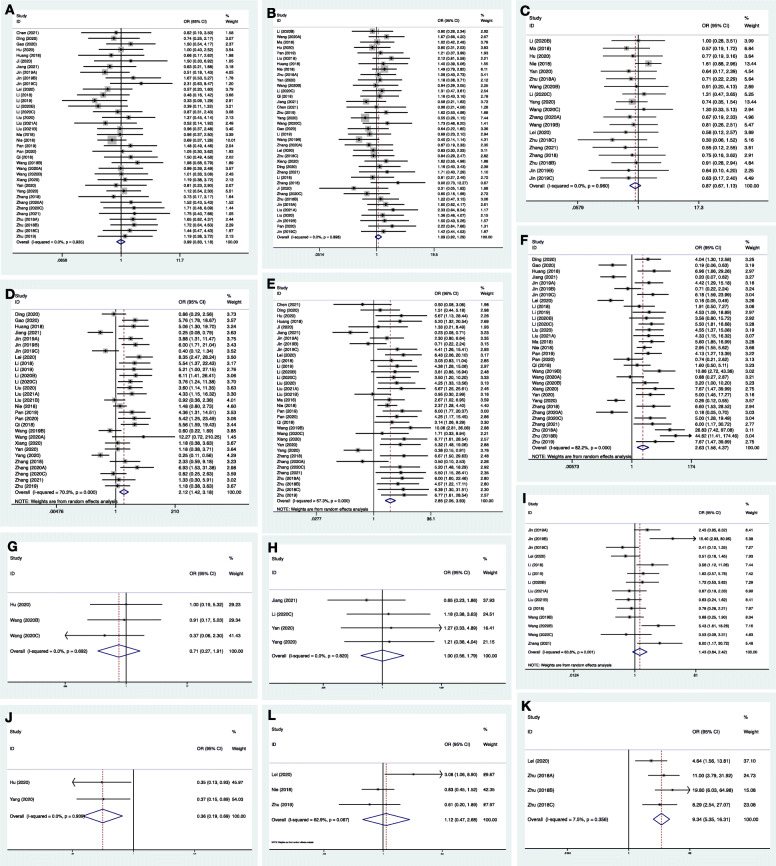
Forest plots evaluated the association between circRNA dysregulation and clinicopathological features of osteosarcoma: (**A**) age, (**B**) gender, (**C**) tumor site, (**D**) tumor size, (**E**) clinical stage, (**F**) metastasis, (**G**) histologic classification, (**H**) histologic pattern, (**I**) differentiation grade, (**J**) chemotherapy response (**K**) chemotherapy resistance, and (**L**) alkaline phosphatase

Table 2 and Fig. 3 show the correlations between circRNAs and clinicopathologic features in 38 selected studies with 2284 osteosarcoma patients. Original data of included studies on clinicopathogical features is summarized in Supplementary Table 3. Dysregulated circRNAs were related to advanced clinicopathologic features, including larger tumor size (OR 2.122, 95%CI 1.418–3.175), advanced clinical stage (OR 2.847, 95%CI 2.059–3.935), and present of metastasis (OR 2.630, 95%CI 1.583–4.371). For chemotherapy, dysregulated circRNAs suggested a better response (OR 0.443, 95%CI 0.231–0.849), but a higher probability of resistance (OR 9.343, 95%CI 5.352–16.309). The heterogeneity of studies on tumor size, clinical stage, metastasis, and chemotherapy response was high. Begg’s and Egger’s tests indicated that studies on tumor size and metastasis have potential high publication bias. The sensitivity analysis showed that the pooled results were stable except for studies on tumor size. The cutoff values of age, tumor size, and clinical stage varied, and corresponding forest plots are presented in Supplementary Fig. 1.

**Table 2:** Pooled odds ratios of circRNAs on clinicopathologic features in osteosarcoma

Clinicopathologic feature	Number of studies	Number of patients	Effect size			Heterogeneity		Sensitivity analysis	Publication bias	
			OR	95%CI	p value	I-square (%)	chi-square (p)		Begg (p)	Egger (p)
Age	37	2239	0.992	0.833–1.181	0.926	0.0%	0.935	Reliable	0.844	0.905
Gender	38	2284	1.086	0.906–1.287	0.342	0.0%	0.898	Reliable	0.297	0.711
Tumor site	19	1229	0.867	0.668–1.125	0.284	0.0%	0.960	Reliable	0.100	**0.003**
Tumor size	29	1749	2.122	1.418–3.175	**< 0.001**	70.3%	< 0.001	Not Reliable	**0.008**	**0.005**
Clinical stage	35	2120	2.847	2.059–3.935	**< 0.001**	57.3%	< 0.001	Reliable	0.191	0.156
Metastasis	32	1975	2.630	1.583–4.371	**< 0.001**	82.2%	<0.001	Reliable	**0.019**	**0.053**
Histologic classification	3	161	0.713	0.266–1.908	0.500	0.0%	0.692	Reliable	0.117	**0.083**
Histologic pattern	4	288	1.000	0.560–1.786	1.000	0.0%	0.820	Reliable	**0.042**	0.228
Differentiation grade	14	737	1.425	0.841–2.415	0.188	63.8%	0.001	Reliable	0.208	0.181
Chemotherapy response	2	158	0.443	0.231–0.849	**0.002**	0.0%	0.554	NA	0.317	NA
Chemotherapy resistance	4	282	9.343	5.352–16.309	**< 0.001**	7.5%	0.365	Reliable	0.497	0.544
Alkaline phosphatase	3	278	1.034	0.648–1.648	0.889	62.9%	0.067	Reliable	0.602	0.743

*CI* confidence interval, *OR* odds ratio

### CircRNAs and prognosis of osteosarcoma

**Fig. 4:**
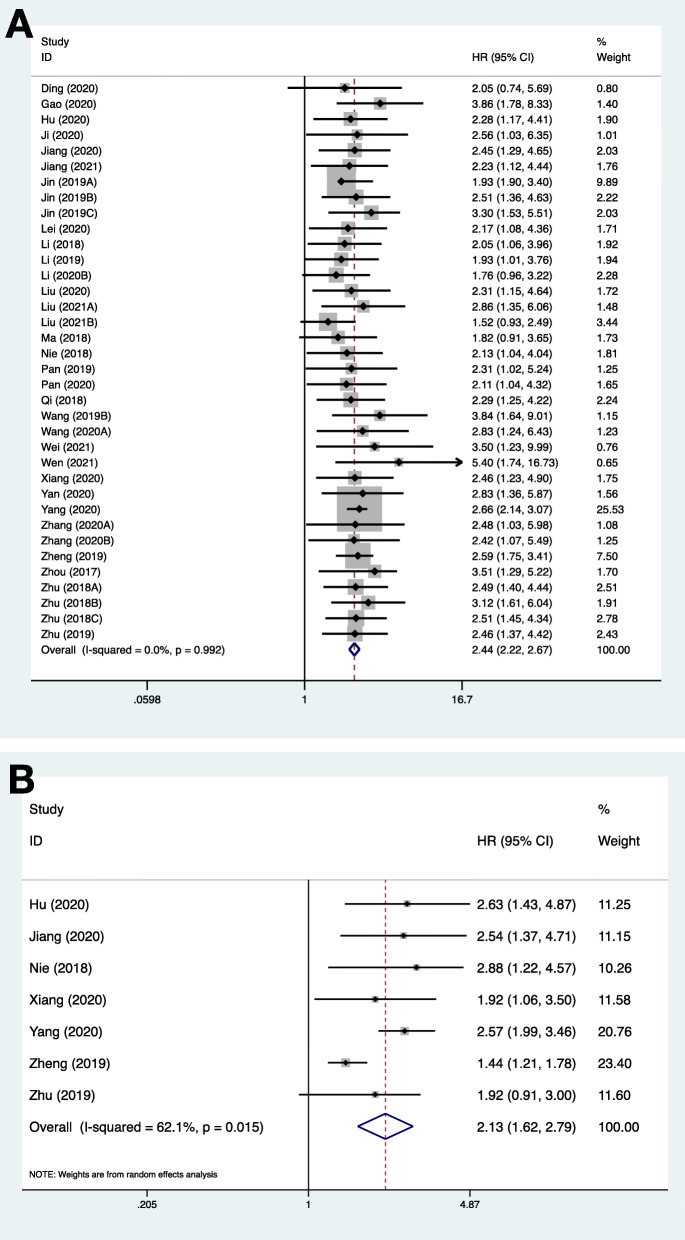
Forest plots assessed the association between circRNA dysregulation and prognosis of osteosarcoma: (**A**) overall survival and (**B**) disease-free survival

**Fig. 5:**
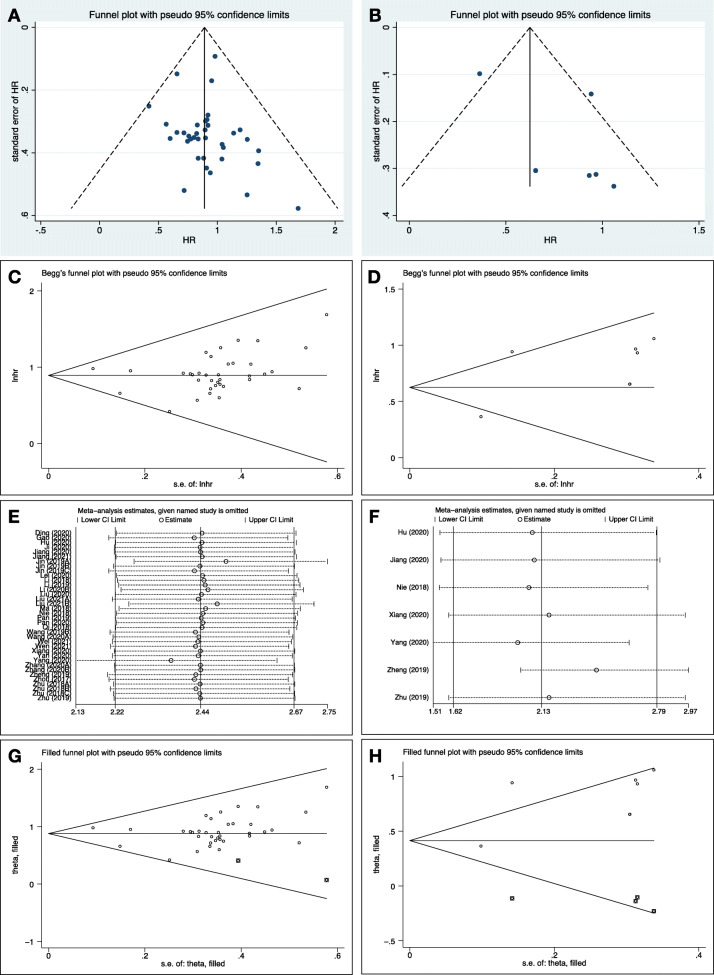
Funnel plots and Begg’s funnel plots judged publication bias of (**A**, **C**) overall survival and (**B**, **D**) disease-free survival in osteosarcoma. Leave-one-out analysis and trim and fill analysis showed the relationship between circRNA dysregulation and prognosis (**E**, **G**) overall survival and (**F**, **H**) disease-free survival of osteosarcoma patients

Table 3 shows the studies on circRNAs and survival prognosis in 44 selected studies, in which 36 studies with 2213 osteosarcoma patients were included in quantitative analysis. Original data of included studies on prognosis is summarized in Supplementary Table 4. Figure 4 and Table 4 present that circRNAs were significantly correlated with OS (HR 2.437, 95%CI 2.224–2.670) with low heterogeneity and reliability. On the other hand, circRNAs were significantly correlated with DFS (HR 2.125, 95%CI 1.621–2.786) with high heterogeneity. Figure 5 reveals the leave-one-out analysis of pooled DFS, indicating that one included study had a significant effect. The funnel plot with Begg’s test and Egger’s test suggested that the likelihood of publication bias was low.

**Table 3:** Survival analysis of circRNAs in osteosarcoma

Author	Year	CircRNA	Regulation pattern	Cutoff	Expression		Survival indicator	Survival analysis	Data availability	Follow-up (month)
					Low	High				
Ding	2020	circ_0005909	Upregulated	Median	27	27	OS	Univariate	K-M curve	60
Gao	2020	circ_0001721	Upregulated	Median	26	30	OS	Univariate	K-M curve (p)	60
Hu	2020	circLARP4	Downregulated	Median	36	36	DFS, OS	Univariate	K-M curve (p)	42
Ji	2020	circ_001621	Upregulated	NR	10	20	OS	Univariate	K-M curve (p)	60
Jiang	2020	circXPO1	Upregulated	Median	26	26	DFS, OS	Univariate	K-M curve (p)	60
Jiang	2021	circ_0000658	Downregulated	Median	30	30	OS	Univariate	K-M curve (p)	60
Jin	2019A	circ_0102049	Upregulated	Median	38	38	OS	Multivariate	Reported (HR)	60
Jin	2019B	circ_100876	Upregulated	Median	24	24	OS	Univariate	K-M curve (p)	60
Jin	2019C	circ_0002052	Downregulated	Median	23	23	OS	Multivariate	Reported (HR)	36
Lei	2020	circ_0003074	Upregulated	Median	36	24	PFS, OS	Univariate	K-M curve (p)	60
Li	2018	circ_0007534	Upregulated	Average	26	31	OS	Multivariate	Reported (HR)	60
Li	2019	circ_0001721	Upregulated	Average	24	28	OS	Multivariate	Reported (HR)	60
Li	2020A	circ_0000073	Upregulated	NR	NR	NR	OS	Univariate	No response	60
Li	2020B	circ 0003732	Upregulated	Median	23	23	OS	Univariate	K-M curve	55
Liu	2020	circ_100284	Upregulated	Median	26	26	OS	Univariate	K-M curve (HR)	125
Liu	2021A	circ_0105346	Upregulated	Median	20	20	OS	Univariate	K-M curve (p)	60
Liu	2021B	circMTO1	Downregulated	NR	32	38	OS	Univariate	K-M curve	60
Ma	2018	circHIPK3	Downregulated	Median	45	37	OS	Univariate	K-M curve	60
Mao	2021	circXPR1	Upregulated	Median	NR	NR	DFS, OS	Univariate	No response	60
Nie	2018	circNT5C2	Upregulated	Median	84	86	DFS, OS	Multivariate	Reported (HR)	60
Pan	2019	circMMP9	Upregulated	NR	27	24	OS	Univariate	K-M curve	60
Pan	2020	circ_103801	Upregulated	NR	18	25	OS	Univariate	K-M curve (p)	60
Qi	2018	circ_0000502	Upregulated	Median	29	34	OS	Multivariate	Reported (HR)	60
Wang	2019A	circ_0003998	Upregulated	NR	NR	NR	OS	Univariate	No response	60
Wang	2019B	circ_0002052	Downregulated	Average	27	33	OS	Multivariate	Reported (HR)	36
Wang	2019C	circ_0021347	Downregulated	NR	NR	NR	OS	Univariate	No response	40
Wang	2020A	circCNST	Upregulated	NR	104	22	OS	Multivariate	Reported (HR)	200
Wei	2021	circ_0081001	Upregulated	Median	31	32	OS	Univariate	K-M curve (p)	60
Wen	2021	circHIPK3	Upregulated	NR	6	6	OS	Univariate	K-M curve (p)	48
Wu	2020	circ_0002052	Downregulated	NR	NR	NR	PFS, OS	Univariate	No response	60
Xiang	2020	circ_0005721	Upregulated	Median	25	25	DFS, OS	Multivariate	K-M curve (HR)	60
Yan	2020	circPVT1	Upregulated	NR	24	24	OS	Univariate	K-M curve (p)	60
Yang	2020	circ_0001105	Upregulated	NR	63	57	DFS, OS	Multivariate	Reported (HR)	60
Zhang	2017	circUBAP2	Upregulated	Median	NR	NR	OS	Univariate	No response	60
Zhang	2019	circ_0051079	Upregulated	NR	NR	NR	OS	Univariate	No response	96
Zhang	2020A	circ_0002052	Upregulated	Median	20	20	OS	Univariate	K-M curve (p)	60
Zhang	2020B	circ_0136666	Upregulated	NR	25	22	OS	Univariate	K-M curve	60
Zhao	2019	circSAMD4A	Upregulated	NR	NR	NR	OS	Univariate	No response	47
Zheng	2019	circLRP6	Upregulated	NR	NR	NR	DFS, OS	Univariate	Reported (HR)	125
Zhou	2017	circ_0008717	Upregulated	ROC	NR	NR	PFS, OS	Multivariate	Reported (HR)	80
Zhu	2018A	circPVT1	Upregulated	Average	50	30	OS	Univariate	K-M curve (p)	60
Zhu	2018B	circ_0081001	Upregulated	Average	55	27	OS	Multivariate	K-M curve (HR)	60
Zhu	2018C	circ_0004674	Upregulated	Average	37	23	OS	Univariate	K-M curve (p)	60
Zhu	2019	circ_0000885	Upregulated	Median	25	25	DFS, OS	Multivariate	K-M curve (HR)	60

*DFS* disease-free survival, *K-M curve* Kaplan-Meier curve, *NA* not applicable, *NR* not reported, *OS* overall survival, *PFS* progression-free survival, *ROC* receiver operation curve analysis

**Table 4:** Pooled hazard ratios of circRNAs on prognosis in osteosarcoma

Prognosis	Number of studies	Number of patients	Effect size			Heterogeneity		Sensitivity analysis	Publication bias	
			HR	95%CI	p value	I-square (%)	chi-square (p)		Begg (p)	Egger (p)
OS	36	2213	2.437	2.224–2.670	< 0.001	0.0%	0.992	Reliable	0.097	0.612
DFS	7	564	2.125	1.621–2.786	< 0.001	62.1%	0.015	Not reliable	0.293	0.136

*CI* confidence interval, *DFS* disease-free survival, *HR* hazard ratio, *OS* overall survival

### Subgroup analysis

Subgroup analysis results of OS can be found in Table 5. All of the subgroups showed a significant correlation between circRNAs and OS of the patients. The results did not show differences among subgroups according to the regulation pattern, sample size, data availability, cutoff value, or NOS. The corresponding forest plots of OS are presented in Supplementary Figure 2.

**Table 5:** Subgroup analysis of overall survival of circRNAs in osteosarcoma

Subgroup	Number of studies	Number of patients	Effect size			Heterogeneity	
			HR	95%CI	p value	I-square (%)	chi-square (p)
Overall	36	3300	2.437	2.224–2.670	< 0.001	0.0%	0.992
Regulation pattern							0.400
Upregulated	30	1823	2.473	2.243–2.726	< 0.001	0.0%	0.998
Downregulated	6	390	2.192	1.684–2.853	< 0.001	11.3%	0.343
Sample size							0.572
≥ 53 samples	18	1411	2.390	2.133–2.678	< 0.001	0.0%	0.806
< 53 samples	18	802	2.525	2.166–2.943	< 0.001	0.0%	0.994
Data availability							0.235
Reported	12	915	2.488	2.209–2.801	< 0.001	0.0%	0.758
K-M curve	7	380	1.882	1.442–2.457	< 0.001	0.0%	0.933
K-M curve (p)	14	734	2.589	2.144–3.126	<0.001	0.0%	0.991
K-M curve (HR)	3	184	2.624	1.769–3.891	< 0.001	0.0%	0.807
Cutoff value							0.482
Median	19	1180	2.279	1.976–2.629	< 0.001	0.0%	0.992
Average	6	391	2.506	1.930–3.256	< 0.001	0.0%	0.797
Other	11	642	2.566	2.245–2.932	< 0.001	0.0%	0.684
NOS score							0.903
≥ 5.5 stars	18	1231	2.457	2.097–2.879	< 0.001	0.0%	0.998
< 5.5 stars	18	982	2.427	2.171–2.714	< 0.001	0.0%	0.715

*CI* confidence interval, *HR* hazard ratio, *K-M curve* Kaplan-Meier curve, *NOS* Newcastle-Ottawa Scale

### Circ_0002052 and osteosarcoma

There were 4 studies repeatably investigated circ_0002052 in osteosarcoma. Table 6 summarizes the 3 available studies with 140 patients and showed that a higher expression of circ_0002052 has a relation with poorer OS (HR 3.197, 95%CI 2.054–4.976). The sensitivity and publication bias analyses have limited significance, since only three studies were included. The corresponding forest plots are presented in Supplementary Figure 3.

**Table 6:** Pooled effect size of circ_0002052 on osteosarcoma

Clinicopathologic and prognostic parameters	Number of studies	Number of patients	Effect size			Heterogeneity		Sensitivity analysis	Publication bias	
			OR/HR	95%CI	p value	I-square (%)	chi-square (p)		Begg (p)	Egger (p)
Age	3	146	1.915	0.959–3.826	0.066	0.0%	0.889	Reliable	0.602	0.944
Gender	3	146	0.697	0.364–1.335	0.276	20.6%	0.284	Reliable	0.602	0.645
Tumor site	3	146	0.709	0.348–1.441	0.342	0.0%	0.960	Reliable	0.117	0.145
Tumor size	3	146	1.101	0.235–5.157	0.903	78.6%	0.009	Not Reliable	0.602	0.387
Clinical stage	3	146	3.016	0.599–15.169	0.181	75.9%	0.016	Not Reliable	0.602	0.249
Differentiation grade	2	106	0.130	0.254–1.192	0.130	0.0%	0.502	NA	0.317	NA
Metastasis	3	146	2.290	0.185–28.348	0.519	90.1%	<0.001	Not Reliable	0.602	0.821
Overall survival	3	146	3.197	2.054–4.976	<0.001	0.0%	0.776	Reliable	0.602	0.825

*CI* confidence interval, *HR* hazard ratio, *OR* odds ratio

## Discussions

Dysregulated circRNA expression has been demonstrated to be important in cancer initiation, development, and immigration [7–9], and has potential as diagnostic and prognostic biomarkers in various tumors [10–12]. Our systematic review conducted a structural literature review and included 52 studies investigating 43 dysregulated circRNAs in 2934 patients with osteosarcoma. We revealed that abnormal circRNA expression was related to tumor size, clinical stage, metastasis, and chemotherapy response and resistance. Further, dysregulated circRNAs were also prognostic biomarkers for OS and DFS. Additionally, dysregulated circ_0002052 was repeatably studied and showed a relation with poorer OS.

Two previous systematic reviews have performed meta-analyses on the clinicopathologic significance and prognostic value of circRNAs in osteosarcoma [21, 22]. The latest review included 31 studies, including 22 on clinicopathologic features and 23 on survival prognosis [22]. Thus, the pooled results may be underpowered due to insufficient data. The review summarized the relation between dysregulated circRNAs and age, gender, tumor size, clinical stage, and metastasis, while our review conducted more analyses on the influence of circRNAs on 12 features with 38 studies. Especially, our analysis on treatment response and resistance provided more practicable insight on treatment decision-making. Moreover, our analysis on survival prognosis included 36 studies to reach more convincing results with increased statistical power. The sensitivity analysis showed the reliability of results that dysregulated circRNAs were promising prognostic biomarkers for osteosarcoma patients. Additionally, our study summarized for the first time that circ_0002052 was significantly correlated with poorer OS with multiple datasets to confirm the efficacy.

Our sensitivity analysis showed that the correlations between dysregulated circRNAs and tumor size and DFS were not reliable, indicating that future studies might change the current results. The publication bias was detected in the analysis of dysregulated circRNAs on tumor size and metastasis, which encouraged more studies on this clinically relevant topic. Subgroup analyses were performed to explore the influence of study characteristics on the pooled results and found that the results remained stable regardless of regulation pattern, sample size, data availability, cutoff value, or study quality, suggesting a potential application in clinical practice.

The quality of included studies was assessed according to the NOS tool, although the overall quality of studies showed a moderate score with a median of 5.5 stars. There were several concerns releveled during our assessment. Most of the included studies put an emphasis on the function of circRNAs in osteosarcoma cells instead of their clinical significance. Therefore, the patient inclusion criteria, treatment procedure, and follow-up were usually unclearly described, which might hinder the clinical translation of circRNAs. The cutoff values were unreported in half of the included studies. Thus, further validation might be impossible. On the other hand, the various cutoff values of clinicopathologic features might introduce a risk of bias into our analysis, including age, tumor size, and clinical stage. To confirm circRNAs as clinically practicable biomarkers, more well-designed and high-quality studies were needed.

The summary of all available circRNAs indicated that circRNAs were significantly correlated with both OS and DFS, while circ_0002052 was the only circRNA that had been studied repeatedly in osteosarcoma patients [41, 58, 65, 72]. The meta-analysis showed that higher expression of circ_0002052 has a relation with poorer OS, but its relation with DFS was not available. Since efficacy confirmed in multiple datasets tends to be more convictive [83], more repeatable and reproducible studies are encouraged to provide more robust evidence for circRNAs as biomarkers for osteosarcoma, to allow translation of circRNAs into clinical practice.

Except for circRNAs, microRNAs and long non-coding RNAs have also shown potential diagnostic, prognostic, and therapeutic values in musculoskeletal malignancies [16–22, 84–86]. On the other hand, evidence is being produced on non-coding RNAs being of importance in benign musculoskeletal diseases [87–90]. These non-coding RNAs could be useful for diagnostic or management purposes in musculoskeletal conditions. However, before they can be applied in clinical practice, the issue of delivery of RNAs needs to be overcome [87, 88].

Our review has several limitations. Firstly, the number of included studies on several clinicopathologic features was comparatively small. Although up to four studies showed that dysregulated circRNA expression has a relation with chemotherapy response and resistance, more studies were encouraged. Secondly, two-thirds of HRs with 95% CIs of OS were indirectly extracted. However, the subgroup analysis demonstrated that there was no significant difference between pooled results according to extraction methods. Thirdly, data from eight studies were impossible to reconstruct, and not available through contraction to the author, which might generate possible bias. Fourthly, the subgroup analysis of DFS was not performed since the number of included studies was limited to draw any stable results. Moreover, we also failed to perform subgroup analyses according to the clinicopathological features of patients, due to varying cutoffs. A more in-depth analysis is encouraged if more future studies provide further details. Fifthly, all of the studies were performed in China, which might lead to biased results due to ethics groups. The role of circRNAs in osteosarcoma among different populations can be evaluated, if investigations in other ethnic groups are available. Finally, only one study obtained circRNA expression data from serum. It is still unclear whether the serum was suitable for circRNA detection in osteosarcoma patients. It might be more practicable and less invasive if the expression detected from serum or plasma had comparable efficiency to those from tissue samples.

## Conclusions

In conclusion, our study showed that there is a significant correlation between the dysregulated expression of circRNAs and advanced clinicopathologic features, and it did affect the survival prognosis of osteosarcoma patients. CircRNAs might play an important role in the occurrence and development of osteosarcoma and showed potential as prognostic biomarkers for osteosarcoma. Our review also pointed out the quality insufficiency in current studies and emphasized the need for prospective high-quality studies with multiple datasets to promote clinical translation.

## Supplementary Information

**Additional file 1.**

## Data Availability

The datasets used and/or analyzed during the current study are available from the corresponding author on reasonable request.

## Abbreviations

ALP: Alkaline phosphatase
CI: Confidence interval
circRNA: Circular ribonucleic acid
HR: Hazard ratio
LDH: Lactate dehydrogenase
OR: Odds ratio
OS: Overall survival
PFS: Progression-free survival
PLR: Positive likelihood ratio
qRT-PCR: Quantitative real-time polymerase chain reaction
NOS: Newcastle-Ottawa Scale
K-M curve: Kaplan-Meier curve
