# Supplementary material for: Clinicopathologic significance and prognostic value of circRNAs in osteosarcoma: a systematic review and meta-analysis
Source: J Orthop Surg Res. 2021 Oct 7;16:578. doi: 10.1186/s13018-021-02568-2 (PMC8495992; doi:10.1186/s13018-021-02568-2)
Supplement: Supplementary file 1 — Additional file 1. [file 13018_2021_2568_MOESM1_ESM.zip › SM-Revision_ESM.docx]

**Supplementary materials**

**Title:** Clinicopathologic significance and prognostic value of circRNAs in osteosarcoma: a systematic review and meta-analysis

**Authors’ name:** Jingyu Zhong^1#^, Guangcheng Zhang^2#^, Weiwu Yao^1*^

# Jingyu Zhong and Guangcheng Zhang have contributed equally to this work; Weiwu Yao is the corresponding author.

**Authors’ affiliations:**

^1^Department of Imaging, Tongren Hospital, Shanghai Jiao Tong University School of Medicine, Shanghai 200336, China

^2^Department of Orthopedics, Shanghai Jiao Tong University Affiliated Sixth People’s Hospital, Shanghai 200233, China

Jingyu Zhong: wal_zjy@163.com (ORCiD: 0000-0002-9817-2294)

Guangcheng Zhang: zgc0922@126.com (ORCiD: 0000-0002-7889-2710)

Weiwu Yao: yaoweiwu@shsmu.edu.cn; yaoweiwuhuan@163.com (ORCiD: 0000-0002-1536-9324)

***Correspondence to**

Prof. Weiwu Yao

Department of Imaging, Tongren Hospital, Shanghai Jiao Tong University School of Medicine, No. 1111 Xianxia Road, Changning District Shanghai 200336, China

Tel: +86-21-52039999

Email: yaoweiwuhuan@163.com

**Content for supplementary materials**

Supplementary Note 1 Search strategy

Supplementary Note 2 Data extraction and reconstruction method

Supplementary Note 3 Data analysis

Supplementary Table 1 Modified NOS tool

Supplementary Table 2 Quality assessment by modified NOS tool

Supplementary Table 3 Original data of included studies on clinicopathogical features

Supplementary Table 4 Original data of included studies on prognosis

Supplementary Figure 1 Forest plots of included studies on clinicopathogical features by cutoff values

Supplementary Figure 2 Forest plots of included studies on overall survival by subgroups

Supplementary Figure 3 Circ_0002052 and osteosarcoma

Supplementary Reference

**Supplementary Note 1 Search strategy**

This search strategy was firstly developed in PubMed according to the research question, and later trailed into other electronic databases and gray literature sources. The search strategy has been tested in a pilot search on 03 Feb 2021 to confirm its feasibility before the formal search. The structured search via electronic databases including PubMed, Embase, The Cochrane Library, Web of Science, Scopus, SinoMed, China National Knowledge Infrastructure (CNKI) and WanFang databases, as well as gray literature sources namely OpenGrey, British Library Inside, ProQuest Dissertations & Theses Global, and BIOSIS preview (via Web of Science), was performed until 20 Feb 2021 by two reviewers independently. Search strategies used are present as following.

**(1) PubMed search strategy**

Avalable via <https://pubmed.ncbi.nlm.nih.gov>

Search date: 03 Feb 2021/20 Feb 2021

Articles retrieved: 132/139

('RNA, Circular'[Mesh] OR circRNA OR ciRNA OR (circular AND RNA) OR 'circular ribonucleic acid') AND ('osteosarcoma'[Mesh] OR osteosarcoma OR (osseous AND sarcoma) OR (osteogenic AND sarcoma))

**(2) Embase search strategy**

Available via <https://www.embase.com>

Search date: 03 Feb 2021/20 Feb 2021

Articles retrieved: 176/182

('circular ribonucleic acid'/exp OR circRNA OR ciRNA OR (circular AND RNA)) AND ('osteosarcoma'/exp OR osteosarcoma OR (osseous AND sarcoma) OR (osteogenic AND sarcoma))

**(3) Cochrane Library search strategy**

Available via <https://www.cochranelibrary.com>

Search date: 03 Feb 2021/20 Feb 2021

Articles retrieved: 0/0

(circRNA OR ciRNA OR (circular AND RNA) OR 'circular ribonucleic acid') AND (osteosarcoma OR (osseous AND sarcoma) OR (osteogenic AND sarcoma))

**(4) Web of Science and BIOSIS preview search strategy**

Available via [apps.webofknowledge.com](http://apps.webofknowledge.com/)

Search date: 03 Feb 2021/20 Feb 2021

Articles retrieved: 187/191

TS=(circRNA OR ciRNA OR (circular AND RNA) OR 'circular ribonucleic acid') AND TS=(osteosarcoma OR (osseous AND sarcoma) OR (osteogenic AND sarcoma))

**(5) Scopus search strategy**

Available via <https://www.scopus.com>

Search date: 03 Feb 2021/20 Feb 2021

Articles retrieved: 72/73

TITLE-ABS-KEY((circRNA OR ciRNA OR (circular AND RNA) OR 'circular ribonucleic acid') AND (osteosarcoma OR (osseous AND sarcoma) OR (osteogenic AND sarcoma)))

**(6) SinoMed search strategy**

Available via <http://www.sinomed.ac.cn>

Search date: 03 Feb 2021/20 Feb 2021

Articles retrieved: 127/131

(circRNA OR ciRNA OR (circular AND RNA) OR 'circular ribonucleic acid' OR 环状RNA) AND (osteosarcoma OR (osseous AND sarcoma) OR (osteogenic AND sarcoma) OR 骨肉瘤)

**(7) CNKI search strategy**

Available via <https://www.cnki.net>

Search date: 03 Feb 2021/20 Feb 2021

Articles retrieved: 207/209

(SU=circRNA OR SU=ciRNA OR SU="circular RNA" OR SU="circular ribonucleic acid" OR SU=环状RAN) AND (SU=osteosarcoma OR SU=(osseous AND sarcoma) OR SU=(osteogenic AND sarcoma) OR SU="骨肉瘤")

**(8) WanFang search strategy**

Available via <http://www.wanfangdata.com.cn>

Search date: 03 Feb 2021/20 Feb 2021

Articles retrieved: 42/43

(circRNA OR ciRNA OR (circular AND RNA) OR 'circular ribonucleic acid' OR 环状RNA) AND (osteosarcoma OR (osseous AND sarcoma) OR (osteogenic AND sarcoma) OR 骨肉瘤)

**(9) OpenGrey search strategy**

Available via <http://www.opengrey.eu>

Search date: 03 Feb 2021/20 Feb 2021

Articles retrieved: 0/0

(circRNA OR ciRNA OR (circular AND RNA) OR 'circular ribonucleic acid') AND (osteosarcoma OR (osseous AND sarcoma) OR (osteogenic AND sarcoma))

**(10) British Library Inside search strategy**

Available via <http://explore.bl.uk/primo_library/libweb/action/search.do>

Search date: 03 Feb 2021/20 Feb 2021

Articles retrieved: 46/54

(circRNA OR ciRNA OR (circular AND RNA) OR 'circular ribonucleic acid') AND (osteosarcoma OR (osseous AND sarcoma) OR (osteogenic AND sarcoma))

**(11) ProQuest Dissertations & Theses Global**

Available via <https://search.proquest.com/pqdtglobal>

Search date: 03 Feb 2021/20 Feb 2021

Articles retrieved: 57/61

ab((circRNA OR ciRNA OR (circular AND RNA) OR 'circular ribonucleic acid') AND (osteosarcoma OR (osseous AND sarcoma) OR (osteogenic AND sarcoma)))

Two reviewers finally included 52 articles [1-52].

**Supplementary Note 2 Data extraction and reconstruction method**

Data extraction was independently completed by two reviewers with our standardized sheet. The data extraction sheet contains following items: (1) bibliographic data: author, publication year, study country; (2) circRNA characteristics: circRNA type, regulation pattern, sample size, specimen type, detection method, cutoff value, number of patients with high or low circRNA expression; (3) clinicopathologic data: age, gender, tumor site, tumor size, clinical stage, histologic classification, differentiation, metastasis; and (4) prognostic information: overall survival (OS), disease-free survival (DFS) or progression-free survival (PFS), hazard ratio (HR) and its 95% confidence interval (CI) for prognostic outcome, analysis method, data availability, follow-up duration. Any disagreement was resolved by discussion or help from a third reviewer.

For clinicopathologic features, all data were extracted directly from the articles. The exposure was defined as higher expression for up-regulated circRNAs in osteosarcoma, and lower expression for down-regulated circRNAs in osteosarcoma, respectively. The outcomes (negative vs positive) were defined as follows: (A) Age: younger vs elder, (B) Gender: female vs male, (C) Tumor site: femur/tibia vs others, (D) Tumor size: smaller vs larger, (E) Clinical stage: lower vs higher, (F) Histologic classification: conventional vs others, (G) Histologic pattern: single component vs mixed, (H) Differentiation grade: lower vs higher, (I) Metastasis: absent vs present, (J) Chemotherapy response: good vs poor, (K) Chemotherapy resistance: not resistant vs resistant, and (L) Alkaline phosphatase: normal vs abnormal.

For prognosis information, we extracted hazard ratio (HR) and its 95% confidence interval (CI) for prognostic outcomes as possible as we can, including overall survival (OS), disease-free survival (DFS) and progression-free survival (PFS). If the studies reported prognostic information in the article, we documented the data directly, otherwise we extracted available data from the Kaplan-Meier curve (K-M curve) via an open-source Engauge Digitizer software version 12.1 [53]. The Engauge Digitizer tool accepts image files (like PNG, JPEG and TIFF) containing graphs, and recovers the data points from those graphs. The resulting data points are usually used as input to other software applications. Conceptually, Engauge Digitizer is the opposite of a graphing tool that converts data points to graphs. An image file is imported and then digitized within Engauge by placing points along axes and curves. Data points can be transferred to other software applications by exporting to a text file, or directly using copy and paste. Work can be saved into an Engauge DIG file for later editing.

Then, we reconstructed the necessary data through several established practical methods for meta-analysis summarized by Tierney JF et al. [54]. They kindly provided an Excel calculator program as supplementary material of their article. For directly reported data, we chose the results of multivariate survival analysis (Cox regression) if performed, otherwise we used the results of univariate survival analysis (Log-rank regression). For articles with HRs and p-values, we reconstructed the 95%CIs based on the HRs and data extracted from the K-M curve; while for those with only p-values, we reconstructed the HRs and their 95%CIs based on the p-values and data extracted from the K-M curve. For articles without HRs or p-values, we redrew the K-M curve to estimate the HRs and their 95%CIs.

The corresponding authors were contacted to request the data, if the articles did not report sufficient data or impossible to reconstruct based on reported data. When there was no response, the article was only qualitatively analyzed.

**Supplementary Note 3 Data analysis**

The characteristics of studies were qualitatively summarized. Total score and those per perspective of NOS were calculated. The statistical analysis was performed with SPSS software version 26.0 (SPSS Inc., Chicago, IL, USA). The meta-analysis was conducted with Stata software version 15.1 (Stata Corp., College Station, TX, USA) using relevant packages. A P-value < 0.05 suggested statistical significance, unless otherwise specified.

For clinicopathogical features, epop stands for patients with exposure and positive outcome, epon stands for patients with exposure and negative outcome, enop stands for patients without exposure and positive outcome, and enon stands for patients without exposure and negative outcome, respectively. Sample size was calculated and summarized. We used ORs and their 95%CIs as estimated effect size. We firstly pooled the studies to assess their heterogeneity. The heterogeneity across studies was measured through the Higgins I-square statistic and chi-square Q test. A random-effect model was applied with the existence of marked heterogeneity as I-square > 50% and chi-square Q p-value < 0.10, otherwise a fixed-effect model was used. For clinicopathogical features with varies cutoffs, analysis by different cutoffs was performed. The publication bias was objectively evaluated by funnel plots and Begg’s funnel plots. Begg’s and Egger’s tests were quantitatively conducted to detect underlying publication bias. A p-value > 0.1 was considered as low publication bias. Sensitivity analysis was used to evaluate the stability of the results. By omitting the included studies one by one, the reliability of the pooled effect size was assessed. A trim and fill method was also used to assess the reliability of results. The code used was preset as follows.

gen samplesize=epop+epon+enop+enon

summarize samplesize, detail

total samplesize

metan epop epon enop enon, label(namevar=author, yearvar=year) [fixed, random] or

metan epop epon enop enon, label(namevar=author, yearvar=year) [fixed, random] or by(cutoffs)

gen log_ES=log(_ES)

metafunnel log_ES _selogES, xtitle(log odds ratio) ytitle(standard error of log OR)

metabias6 log_ES _selogES, graph(begg)

metaninf epop epon enop enon, label(namevar=author, yearvar=year) [fixed, random] or

metatrim log_ES _selogES, eform funnel

For prognosis, we translated the HRs and 95%CI into a form that HRs >1 suggested poor prognosis and was considered statistically significant if the 95%CI did not contain 1. The heterogeneity was assessed through the Higgins I-square statistic and chi-square Q test. A random-effect model was applied with the existence of marked heterogeneity as I-square > 50% and chi-square Q p-value < 0.10, otherwise a fixed-effect model was used. The publication bias was objectively evaluated by funnel plots and Begg’s funnel plots. Begg’s and Egger’s tests were quantitatively conducted to detect underlying publication bias. A p-value > 0.1 was considered as low publication bias. Sensitivity analysis was used to evaluate the stability of the results. By omitting the included studies one by one, the reliability of the pooled effect size was assessed. A trim and fill method was also used to assess the reliability of results. The code used was preset as follows.

summarize samplesize, detail

total samplesize

gen lnhr=ln(hr)

gen lnll=ln(lower95ci)

gen inul=ln(upper95ci)

metan lnhr lnll lnul, [fixed, random] eform label(namevar=author, yearvar=year) effect(HR)

metafunnel hr lower95ci upper95ci, xtitle(HR)ytitle(standard error of HR)

gen selnhr=(lnul-lnll)/3.92

metabias6 lnhr selnhr,graph(begg)

metaninf lnhr selnhr, [fixed, random] label(namevar=author,yearvar=year)eform

metatrim lnhr selnhr, eform funnel

Subgroup analyses were performed to explore potential sources of heterogeneity, according to (1) regulation pattern: up-regulated, or down-regulated; (2) sample size: < 53 samples (median), or ≥ 53 samples; (3) data availability: reported or K-M curve; and (4) cut-off value: median, average, or others; and (5) NOS: score < 5.5 (median), score ≥ 5.5. The code used was preset as follows.

metan lnhr lnll lnul, fixed eform label(namevar=author, yearvar=year) effect(HR)by(regulationpattern)

gen ss53=1 if samplesize>=53

replace ss53=0 if samplesize<53

metan lnhr lnll lnul, fixed eform label(namevar=author, yearvar=year) effect(HR)by(ss53)

metan lnhr lnll lnul, fixed eform label(namevar=author, yearvar=year) effect(HR)by(dataavailability)

metan lnhr lnll lnul, fixed eform label(namevar=author, yearvar=year) effect(HR)by(cutoff)

gen nos55=1 if nos>=5.5

replace nos55=0 if nos<5.5

metan lnhr lnll lnul, fixed eform label(namevar=author, yearvar=year) effect(HR)by(nos55)

**Supplementary Table 1 Modified NOS tool**

| Item | Original description | Modified version |
| --- | --- | --- |
| Case-control study | | |
| Selection 1 | **Is the case definition adequate?**  a) yes, with independent validation (*);  b) yes, e.g. record linkage or based on self-reports;  c) no description | The cases were defined as higher group in up-regulated circRNAs, while lower group in down-regulated circRNAs. If the dysregulation pattern of circRNAs confirmed by osteosarcoma cells lines or bioinformatic analysis, the definition of cases was considered to be adequate.  a) dysregulation pattern of circRNAs confirmed by osteosarcoma cells lines or bioinformatic analysis (*);  b) no description |
| Selection 2 | **Representativeness of the cases**  a) consecutive or obviously representative series of cases (*);  b) potential for selection biases or not stated; | If the study included the patients in a consecutive manner, the cases were considered to be representative.  a) consecutive inclusion of patients (*);  b) no description |
| Selection 3 | **Selection of controls**  a) community controls (*);  b) hospital controls;  c) no description | Since all the included samples were osteosarcoma patients, community controls were not available. We considered the controls to be representative, if the patients selected from the same resource.  a) the same resource (*)  b) no description |
| Selection 4 | **Definition of controls**  a) no history of disease (endpoint) (*);  b) no description of source | The controls were defined as lower group in up-regulated circRNAs, while higher group in down-regulated circRNAs. qRT-PCR was considered as an adequate independent validation for circRNA expression.  a) cutoff described (*);  b) no description |
| Comparability | **Comparability of cases and controls on the basis of the design or analysis**  a) study controls for _______________ (Select the most important factor.) (*);  b) study controls for any additional factor (This criterion could be modified to indicate specific  control for a second important factor.) (*) | If the study treated the patients with similar therapy, the cases and controls were considered to be comparable. If the study described the comparable qRT-PCR protocol in cases and controls, an additional star was given.  a) similar therapy (*);  b) qRT-PCR protocol described (*) |
| Exposure 1 | **Ascertainment of exposure**  a) secure record (e.g. surgical records) (*);  b) structured interview where blind to case/control status (*);  c) interview not blinded to case/control status;  d) written self-report or medical record only;  e) no description | qRT-PCR was considered as a secure record for circRNA expression. Other detection methods were not suitable.  a) qRT-PCR was used to detected the circRNA expression (*);  b) no description |
| Exposure 2 | **Same method of ascertainment for cases and controls**  a) yes (*);  b) no | The circRNA expression of cases and controls should be detected through comparable qRT-PCR protocol.  a) the same qRT-PCR protocol used (*);  b) no description |
| Exposure 3 | **Non-response rate**  a) same rate for both groups (*);  b) non respondents described;  c) rate different and no designation | The response rate was considered to be the same, if the reported all the outcome results were available in the study.  a) same rate for both groups (*);  b) rate different and no designation |
| Cohort study | | |
| Selection 1 | **Representativeness of the exposed cohort**  a) truly representative of the average _______________ (describe) in the community (*);  b) somewhat representative of the average ______________ in the community (*);  c) selected group of users e.g. nurses, volunteers;  d) no description of the derivation of the cohort. | If the study included the patients in a consecutive manner, the cohort was considered to be representative.  a) consecutive inclusion of patients (*);  b) no description |
| Selection 2 | **Selection of the non-exposed cohort**  a) drawn from the same community as the exposed cohort (*);  b) drawn from a different source;  c) no description of the derivation of the non-exposed cohort | Since all the included samples were osteosarcoma patients, community controls were not available. We considered the controls to be representative., if the study reported the cutoff for subgrouping.  a) the cutoff was reported (*);  b) the cutoff was not reported |
| Selection 3 | **Ascertainment of exposure**  a) secure record (e.g. surgical records) (*);  b) structured interview (*);  c) written self-report;  d) no description | qRT-PCR was considered as an adequate independent validation for circRNA expression.  a) qRT-PCR was used to detected the circRNA expression (*);  b) no description |
| Selection 4 | **Demonstration that outcome of interest was not present at start of study**  a) yes (*);  b) no | The outcome was overall or disease-free survival in prognosis studies. The outcome of interest was considered to be not present at start of follow-up, if an adequate treatment procedure was clearly described.  a) treatment procedure was clearly described (*);  b) unclear or inadequate treatment procedure or not reported |
| Comparability | **Comparability of cohorts on the basis of the design or analysis**  a) study controls for _____________ (select the most important factor) (*);  b) study controls for any additional factor (This criterion could be modified to indicate specific  control for a second important factor.) (*) | If the study used a multivariate survival analysis, a star was given. If the study described the comparable qRT-PCR protocol in cases and controls, an additional star was given.  a) multivariate survival analysis (*);  b) qRT-PCR protocol described (*) |
| Outcome 1 | **Assessment of outcome**  a) independent blind assessment (*);  b) record linkage (*);  c) self-report;  d) no description | The outcome assessment was considered to be unlikely to introduce bias, if the check-up plan was described in the study.  a) check-up plan described (*);  b) check-up plan not described |
| Outcome 2 | **Was follow-up long enough for outcomes to occur**  a) yes (select an adequate follow up period for outcome of interest) (*);  b) no | The follow-up duration was considered to be adequate, if the period was 60 months or longer.  a) follow-up duration≥ 60 months (*);  b) follow-up duration< 60 months |
| Outcome 3 | **Adequacy of follow up of cohorts**  a) complete follow up - all subjects accounted for (*);  b) subjects lost to follow up unlikely to introduce bias - small number lost - > ____ % (select an  adequate %) follow up, or description provided of those lost) (*);  c) follow up rate < ____% (select an adequate %) and no description of those lost;  d) no statement | The subjects lost to follow up was considered to be unlikely to introduce bias, if follow-up rate was≥ 90%.  a) follow-up rate ≥ 90% (*);  b) follow-up rate < 90% or not reported |

Abbreviations: NOS = Newcastle - Ottawa Scale

**Supplementary Table 2 Quality assessment by modified NOS tool**

| Author | Year | Outcome | Selection | | | | Comparability | Exposure/Outcome | | | Total | Ref |
| --- | --- | --- | --- | --- | --- | --- | --- | --- | --- | --- | --- | --- |
|  |  |  | 1 | 2 | 3 | 4 |  | 1 | 2 | 3 |  |  |
| Chen | 2021 | CP | 1 | 0 | 1 | 0 | 1 | 1 | 1 | 0 | 5 | [1] |
| Ding | 2020 | CP, OS | 0 | 1 | 1 | 1 | 1 | 0 | 1 | 0 | 5 | [2] |
| Gao | 2020 | CP, OS | 0 | 1 | 1 | 0 | 1 | 0 | 1 | 0 | 4 | [3] |
| Hu | 2020 | CP, DFS, OS | 1 | 1 | 1 | 1 | 1 | 1 | 0 | 0 | 6 | [4] |
| Huang | 2018 | CP | 1 | 0 | 1 | 0 | 1 | 1 | 1 | 1 | 6 | [5] |
| Ji | 2020 | CP, OS | 1 | 0 | 1 | 1 | 1 | 0 | 1 | 1 | 6 | [6] |
| Jiang | 2020 | DFS, OS | 0 | 1 | 1 | 1 | 1 | 0 | 1 | 0 | 5 | [7] |
| Jiang | 2021 | CP, OS | 0 | 1 | 1 | 0 | 1 | 0 | 1 | 0 | 4 | [8] |
| Jin | 2019A | CP, OS | 0 | 1 | 1 | 0 | 2 | 0 | 1 | 0 | 5 | [9] |
| Jin | 2019B | CP, OS | 1 | 1 | 1 | 0 | 1 | 0 | 1 | 0 | 5 | [10] |
| Jin | 2019C | CP, OS | 1 | 1 | 1 | 0 | 2 | 1 | 0 | 0 | 6 | [11] |
| Lei | 2020 | CP, DFS, OS | 1 | 1 | 1 | 1 | 1 | 0 | 1 | 0 | 6 | [12] |
| Li | 2018 | CP, OS | 1 | 1 | 1 | 0 | 2 | 0 | 1 | 0 | 6 | [13] |
| Li | 2019 | CP, OS | 1 | 1 | 1 | 0 | 2 | 0 | 1 | 0 | 6 | [14] |
| Li | 2020A | OS | 1 | 0 | 1 | 1 | 1 | 0 | 1 | 0 | 5 | [15] |
| Li | 2020B | CP, OS | 0 | 1 | 1 | 1 | 1 | 0 | 0 | 0 | 4 | [16] |
| Li | 2020C | CP | 1 | 0 | 1 | 0 | 1 | 1 | 1 | 1 | 6 | [17] |
| Liu | 2020 | CP, OS | 0 | 1 | 1 | 0 | 1 | 0 | 1 | 0 | 4 | [18] |
| Liu | 2021A | CP, OS | 0 | 1 | 1 | 1 | 1 | 1 | 1 | 0 | 6 | [19] |
| Liu | 2021B | CP, OS | 1 | 0 | 1 | 1 | 1 | 0 | 1 | 0 | 5 | [20] |
| Ma | 2018 | CP, OS | 1 | 1 | 1 | 1 | 1 | 0 | 1 | 0 | 6 | [21] |
| Mao | 2021 | DFS, OS | 1 | 1 | 1 | 0 | 1 | 0 | 1 | 0 | 5 | [22] |
| Nie | 2018 | CP, DFS, OS | 0 | 1 | 1 | 1 | 2 | 0 | 1 | 1 | 7 | [23] |
| Pan | 2019 | CP, OS | 0 | 0 | 1 | 1 | 1 | 0 | 1 | 0 | 4 | [24] |
| Pan | 2020 | CP, OS | 0 | 0 | 1 | 0 | 1 | 0 | 1 | 0 | 3 | [25] |
| Qi | 2018 | CP, OS | 1 | 1 | 1 | 0 | 2 | 0 | 1 | 0 | 6 | [26] |
| Wang | 2019A | OS | 1 | 0 | 1 | 1 | 1 | 0 | 1 | 0 | 5 | [27] |
| Wang | 2019B | CP, OS | 1 | 1 | 1 | 1 | 2 | 1 | 0 | 0 | 7 | [28] |
| Wang | 2019C | OS | 1 | 0 | 1 | 0 | 1 | 0 | 0 | 0 | 3 | [29] |
| Wang | 2020A | CP, OS | 1 | 0 | 1 | 1 | 2 | 0 | 1 | 0 | 6 | [30] |
| Wang | 2020B | CP | 1 | 0 | 1 | 0 | 1 | 1 | 1 | 1 | 6 | [31] |
| Wang | 2020C | CP | 1 | 0 | 1 | 0 | 1 | 1 | 1 | 1 | 6 | [32] |
| Wei | 2021 | OS | 0 | 1 | 1 | 1 | 1 | 0 | 1 | 0 | 5 | [33] |
| Wen | 2021 | OS | 1 | 0 | 1 | 0 | 1 | 0 | 0 | 0 | 3 | [34] |
| Wu | 2020 | PFS, OS | 0 | 0 | 1 | 0 | 1 | 0 | 1 | 0 | 3 | [35] |
| Xiang | 2020 | CP, DFS, OS | 1 | 1 | 1 | 1 | 2 | 1 | 1 | 0 | 8 | [36] |
| Yan | 2020 | CP, OS | 1 | 0 | 1 | 0 | 1 | 0 | 1 | 0 | 4 | [37] |
| Yang | 2020 | CP, DFS, OS | 0 | 0 | 1 | 1 | 2 | 0 | 1 | 0 | 5 | [38] |
| Zhang | 2017 | OS | 0 | 1 | 1 | 0 | 1 | 0 | 1 | 0 | 4 | [39] |
| Zhang | 2018 | CP | 1 | 1 | 1 | 0 | 2 | 1 | 1 | 1 | 8 | [40] |
| Zhang | 2019 | OS | 1 | 0 | 1 | 0 | 1 | 0 | 1 | 0 | 4 | [41] |
| Zhang | 2020A | CP, OS | 0 | 1 | 1 | 0 | 1 | 0 | 1 | 0 | 4 | [42] |
| Zhang | 2020B | OS | 0 | 0 | 1 | 0 | 1 | 0 | 1 | 0 | 3 | [43] |
| Zhang | 2020C | CP | 1 | 0 | 1 | 1 | 1 | 1 | 1 | 1 | 7 | [44] |
| Zhang | 2021 | CP | 1 | 1 | 1 | 0 | 1 | 1 | 1 | 1 | 7 | [45] |
| Zhao | 2019 | OS | 1 | 0 | 1 | 0 | 1 | 0 | 0 | 0 | 3 | [46] |
| Zheng | 2019 | DFS, OS | 1 | 0 | 1 | 0 | 1 | 0 | 1 | 0 | 4 | [47] |
| Zhou | 2017 | PFS, OS | 1 | 1 | 1 | 0 | 2 | 0 | 1 | 0 | 6 | [48] |
| Zhu | 2018A | CP, OS | 1 | 1 | 1 | 1 | 1 | 0 | 1 | 0 | 6 | [49] |
| Zhu | 2018B | CP, OS | 1 | 1 | 1 | 1 | 2 | 0 | 1 | 0 | 7 | [50] |
| Zhu | 2018C | CP, OS | 1 | 1 | 1 | 1 | 1 | 0 | 1 | 0 | 6 | [51] |
| Zhu | 2019 | CP, DFS, OS | 0 | 1 | 1 | 1 | 2 | 0 | 1 | 0 | 6 | [52] |

Abbreviations: NOS = Newcastle - Ottawa Scale

**Supplementary Table 3 Original data of included studies on clinicopathogical features**

| Author | Year | CircRNA | Regulation pattern | Outcome | Cutoff (- vs +) | P value | E+O+ | E+O- | E-O+ | E-O- | Ref |
| --- | --- | --- | --- | --- | --- | --- | --- | --- | --- | --- | --- |
| Chen | 2021 | circ_0000885 | up-regulated | Age | <50 vs ≥50 y/o | 0.210 | 7 | 10 | 6 | 7 | [1] |
| Ding | 2020 | circ_0005909 | up-regulated | Age | <18 vs ≥18 y/o | 0.586 | 12 | 15 | 14 | 13 | [2] |
| Gao | 2020 | circ_0001721 | up-regulated | Age | ≤60 vs >60 y/o | 0.436 | 15 | 15 | 12 | 18 | [3] |
| Hu | 2020 | circLARP4 | down-regulated | Age | <18 vs ≥18 y/o | 1.000 | 17 | 19 | 17 | 19 | [4] |
| Huang | 2018 | circNASP | up-regulated | Age | <25 vs ≥25 y/o | 0.731 | 5 | 14 | 7 | 13 | [5] |
| Ji | 2020 | circ_001621 | up-regulated | Age | <25 vs ≥25 y/o | 0.602 | 12 | 8 | 5 | 5 | [6] |
| Jiang | 2021 | circ_0000658 | down-regulated | Age | <18 vs ≥18 y/o | 0.5796 | 8 | 22 | 11 | 19 | [8] |
| Jin | 2019A | circ_0102049 | up-regulated | Age | <30 vs ≥30 y/o | 0.305 | 8 | 30 | 13 | 25 | [9] |
| Jin | 2019B | circ_100876 | up-regulated | Age | <20 vs ≥20 y/o | 0.383 | 15 | 9 | 12 | 12 | [10] |
| Jin | 2019C | circ_0002052 | down-regulated | Age | ≤25 vs >25 y/o | 0.200 | 18 | 5 | 14 | 9 | [11] |
| Lei | 2020 | circ_0003074 | up-regulated | Age | <25 vs ≥25 y/o | 0.314 | 11 | 18 | 16 | 15 | [12] |
| Li | 2018 | circ_0007534 | up-regulated | Age | <40 vs ≥40 y/o | 0.271 | 9 | 22 | 12 | 14 | [13] |
| Li | 2019 | circ_0001721 | up-regulated | Age | <40 vs ≥40 y/o | 0.186 | 4 | 24 | 8 | 16 | [14] |
| Li | 2020B | circ 0003732 | up-regulated | Age | <25 vs ≥25 y/o | 0.111 | 6 | 17 | 11 | 12 | [16] |
| Li | 2020C | circ_0000190 | down-regulated | Age | <18 vs ≥18 y/o | 1.000 | 12 | 18 | 13 | 17 | [17] |
| Liu | 2020 | circ_100284 | up-regulated | Age | ≤20 vs >20 y/o | 0.5745 | 12 | 14 | 10 | 16 | [18] |
| Liu | 2021A | circ_0105346 | up-regulated | Age | ≤20 vs >20 y/o | 0.327 | 6 | 14 | 9 | 11 | [19] |
| Liu | 2021B | circMTO1 | down-regulated | Age | <18 vs ≥18 y/o | 0.934 | 21 | 17 | 18 | 14 | [20] |
| Ma | 2018 | circHIPK3 | down-regulated | Age | <25 vs ≥25 y/o | 0.63 | 13 | 32 | 11 | 26 | [21] |
| Nie | 2018 | circNT5C2 | up-regulated | Age | <25 vs ≥25 y/o | 0.565 | 37 | 49 | 44 | 40 | [23] |
| Pan | 2019 | circMMP9 | up-regulated | Age | <20 vs ≥20 y/o | 0.5793 | 13 | 11 | 12 | 15 | [24] |
| Pan | 2020 | circ_103801 | up-regulated | Age | <25 vs ≥25 y/o | 0.9414 | 10 | 15 | 7 | 11 | [25] |
| Qi | 2018 | circ_0000502 | up-regulated | Age | <40 vs ≥40 y/o | 0.580 | 11 | 23 | 7 | 22 | [26] |
| Wang | 2019B | circ_0002052 | down-regulated | Age | ≤25 vs >25 y/o | 0.210 | 19 | 8 | 18 | 15 | [28] |
| Wang | 2020A | circCNST | up-regulated | Age | <60 vs ≥60 y/o | 1.000 | 12 | 10 | 57 | 47 | [30] |
| Wang | 2020B | circTCF25 | up-regulated | Age | <18 vs ≥18 y/o | 0.789 | 12 | 14 | 11 | 13 | [31] |
| Xiang | 2020 | circ_0005721 | up-regulated | Age | <25 vs ≥25 y/o | 0.500 | 10 | 15 | 9 | 16 | [36] |
| Yan | 2020 | circPVT1 | up-regulated | Age | <18 vs ≥18 y/o | 0.745 | 6 | 18 | 7 | 17 | [37] |
| Yang | 2020 | circ_0001105 | up-regulated | Age | ≤18 vs >18 y/o | 0.905 | 26 | 31 | 27 | 36 | [38] |
| Zhang | 2018 | circ_001569 | up-regulated | Age | ≤18 vs >18 y/o | 0.667 | 5 | 15 | 5 | 11 | [40] |
| Zhang | 2020A | circ_0002052 | up-regulated | Age | <18 vs ≥18 y/o | 0.7475 | 9 | 11 | 7 | 13 | [42] |
| Zhang | 2020C | circ_0017247 | up-regulated | Age | ≤18 vs >18 y/o | 0.403 | 8 | 14 | 6 | 18 | [44] |
| Zhang | 2021 | circ_0005909 | up-regulated | Age | <18 vs ≥18 y/o | 0.456 | 7 | 8 | 5 | 10 | [45] |
| Zhu | 2018A | circPVT1 | up-regulated | Age | <25 vs ≥25 y/o | 0.63 | 11 | 19 | 13 | 37 | [49] |
| Zhu | 2018B | circ_0081001 | up-regulated | Age | <25 vs ≥25 y/o | 0.279 | 10 | 17 | 14 | 41 | [50] |
| Zhu | 2018C | circ_0004674 | up-regulated | Age | <25 vs ≥25 y/o | 0.26 | 8 | 15 | 10 | 27 | [51] |
| Zhu | 2019 | circ_0000885 | up-regulated | Age | <25 vs ≥25 y/o | 0.500 | 10 | 15 | 9 | 16 | [52] |
| Chen | 2021 | circ_0000885 | up-regulated | Gender | F vs M | 0.364 | 12 | 7 | 7 | 4 | [1] |
| Ding | 2020 | circ_0005909 | up-regulated | Gender | F vs M | 0.783 | 16 | 11 | 15 | 12 | [2] |
| Gao | 2020 | circ_0001721 | up-regulated | Gender | F vs M | 0.410 | 14 | 16 | 15 | 11 | [3] |
| Hu | 2020 | circLARP4 | down-regulated | Gender | F vs M | 0.633 | 20 | 16 | 22 | 14 | [4] |
| Huang | 2018 | circNASP | up-regulated | Gender | F vs M | 0.748 | 12 | 7 | 11 | 9 | [5] |
| Ji | 2020 | circ_001621 | up-regulated | Gender | F vs M | 0.401 | 11 | 9 | 8 | 2 | [6] |
| Jiang | 2021 | circ_0000658 | down-regulated | Gender | F vs M | 0.4348 | 11 | 19 | 15 | 15 | [8] |
| Jin | 2019A | circ_0102049 | up-regulated | Gender | F vs M | 0.469 | 27 | 11 | 23 | 15 | [9] |
| Jin | 2019B | circ_100876 | up-regulated | Gender | F vs M | 0.525 | 18 | 6 | 16 | 8 | [10] |
| Jin | 2019C | circ_0002052 | down-regulated | Gender | F vs M | 0.555 | 13 | 10 | 11 | 12 | [11] |
| Lei | 2020 | circ_0003074 | up-regulated | Gender | F vs M | 0.293 | 18 | 14 | 17 | 11 | [12] |
| Li | 2018 | circ_0007534 | up-regulated | Gender | F vs M | 0.782 | 20 | 11 | 18 | 8 | [13] |
| Li | 2019 | circ_0001721 | up-regulated | Gender | F vs M | 0.581 | 15 | 13 | 15 | 9 | [14] |
| Li | 2020B | circ 0003732 | up-regulated | Gender | F vs M | 0.277 | 14 | 19 | 11 | 12 | [16] |
| Li | 2020C | circ_0000190 | down-regulated | Gender | F vs M | 0.7961 | 15 | 15 | 13 | 17 | [17] |
| Liu | 2020 | circ_100284 | up-regulated | Gender | F vs M | 0.5780 | 15 | 11 | 13 | 13 | [18] |
| Liu | 2021A | circ_0105346 | up-regulated | Gender | F vs M | 0.197 | 14 | 6 | 10 | 10 | [19] |
| Liu | 2021B | circMTO1 | down-regulated | Gender | F vs M | 0.126 | 20 | 18 | 11 | 21 | [20] |
| Ma | 2018 | circHIPK3 | down-regulated | Gender | F vs M | 0.84 | 27 | 18 | 22 | 15 | [21] |
| Nie | 2018 | circNT5C2 | up-regulated | Gender | F vs M | 0.215 | 60 | 26 | 51 | 33 | [23] |
| Pan | 2019 | circMMP9 | up-regulated | Gender | F vs M | 0.7724 | 17 | 7 | 18 | 9 | [24] |
| Pan | 2020 | circ_103801 | up-regulated | Gender | F vs M | 0.2027 | 16 | 9 | 8 | 10 | [25] |
| Qi | 2018 | circ_0000502 | up-regulated | Gender | F vs M | 0.803 | 20 | 14 | 16 | 13 | [26] |
| Wang | 2019B | circ_0002052 | down-regulated | Gender | F vs M | 0.08 | 12 | 15 | 22 | 11 | [28] |
| Wang | 2020A | circCNST | up-regulated | Gender | F vs M | 0.339 | 11 | 11 | 39 | 65 | [30] |
| Wang | 2020B | circTCF25 | up-regulated | Gender | F vs M | 0.934 | 17 | 9 | 16 | 8 | [31] |
| Wang | 2020C | circ_0001658 | up-regulated | Gender | F vs M | 0.3994 | 11 | 10 | 7 | 11 | [32] |
| Xiang | 2020 | circ_0005721 | up-regulated | Gender | F vs M | 0.285 | 15 | 10 | 12 | 13 | [36] |
| Yan | 2020 | circPVT1 | up-regulated | Gender | F vs M | 0.771 | 11 | 13 | 10 | 14 | [37] |
| Yang | 2020 | circ_0001105 | up-regulated | Gender | F vs M | 0.160 | 19 | 38 | 30 | 33 | [38] |
| Zhang | 2018 | circ_001569 | up-regulated | Gender | F vs M | 0.121 | 15 | 5 | 8 | 8 | [40] |
| Zhang | 2020A | circ_0002052 | up-regulated | Gender | F vs M | 0.7512 | 8 | 12 | 10 | 10 | [42] |
| Zhang | 2020C | circ_0017247 | up-regulated | Gender | F vs M | 0.400 | 12 | 10 | 16 | 8 | [44] |
| Zhang | 2021 | circ_0005909 | up-regulated | Gender | F vs M | 0.464 | 9 | 6 | 7 | 8 | [45] |
| Zhu | 2018A | circPVT1 | up-regulated | Gender | F vs M | 0.84 | 18 | 12 | 29 | 21 | [49] |
| Zhu | 2018B | circ_0081001 | up-regulated | Gender | F vs M | 0.678 | 17 | 10 | 32 | 23 | [50] |
| Zhu | 2018C | circ_0004674 | up-regulated | Gender | F vs M | 0.44 | 14 | 9 | 24 | 13 | [51] |
| Zhu | 2019 | circ_0000885 | up-regulated | Gender | F vs M | 0.285 | 15 | 10 | 12 | 13 | [52] |
| Hu | 2020 | circLARP4 | down-regulated | Tumor site | Femur/Tibia vs Others | 0.750 | 4 | 32 | 5 | 31 | [4] |
| Jin | 2019B | circ_100876 | up-regulated | Tumor site | Femur/Tibia vs Others | 0.888 | 2 | 22 | 3 | 21 | [10] |
| Jin | 2019C | circ_0002052 | down-regulated | Tumor site | Femur/Tibia vs Others | 0.502 | 5 | 18 | 7 | 16 | [11] |
| Lei | 2020 | circ_0003074 | up-regulated | Tumor site | Femur/Tibia vs Others | 0.308 | 3 | 27 | 5 | 25 | [12] |
| Li | 2020B | circ 0003732 | up-regulated | Tumor site | Femur/Tibia vs Others | 0.625 | 7 | 16 | 7 | 16 | [16] |
| Li | 2020C | circ_0000190 | down-regulated | Tumor site | Femur/Tibia vs Others | 0.6023 | 14 | 16 | 12 | 18 | [17] |
| Ma | 2018 | circHIPK3 | down-regulated | Tumor site | Femur/Tibia vs Others | 0.45 | 7 | 38 | 9 | 28 | [21] |
| Nie | 2018 | circNT5C2 | up-regulated | Tumor site | Femur/Tibia vs Others | 0.121 | 45 | 41 | 34 | 50 | [23] |
| Wang | 2019B | circ_0002052 | down-regulated | Tumor site | Femur/Tibia vs Others | 0.49 | 7 | 20 | 10 | 23 | [28] |
| Wang | 2020B | circTCF25 | up-regulated | Tumor site | Femur/Tibia vs Others | 0.587 | 4 | 22 | 4 | 20 | [31] |
| Wang | 2020C | circ_0001658 | up-regulated | Tumor site | Femur/Tibia vs Others | 0.4475 | 7 | 14 | 5 | 13 | [32] |
| Yan | 2020 | circPVT1 | up-regulated | Tumor site | Femur/Tibia vs Others | 0.505 | 5 | 19 | 7 | 17 | [37] |
| Yang | 2020 | circ_0001105 | up-regulated | Tumor site | Femur/Tibia vs Others | 0.019 | 33 | 24 | 41 | 22 | [38] |
| Zhang | 2018 | circ_001569 | up-regulated | Tumor site | Femur/Tibia vs Others | 0.799 | 4 | 16 | 4 | 12 | [40] |
| Zhang | 2020A | circ_0002052 | up-regulated | Tumor site | Femur/Tibia vs Others | 0.7524 | 9 | 11 | 11 | 9 | [42] |
| Zhang | 2021 | circ_0005909 | up-regulated | Tumor site | Femur/Tibia vs Others | 0.439 | 9 | 6 | 11 | 4 | [45] |
| Zhu | 2018A | circPVT1 | up-regulated | Tumor site | Femur/Tibia vs Others | 0.45 | 5 | 25 | 11 | 39 | [49] |
| Zhu | 2018B | circ_0081001 | up-regulated | Tumor site | Femur/Tibia vs Others | 0.928 | 5 | 22 | 11 | 44 | [50] |
| Zhu | 2018C | circ_0004674 | up-regulated | Tumor site | Femur/Tibia vs Others | 0.55 | 2 | 21 | 9 | 28 | [51] |
| Ding | 2020 | circ_0005909 | up-regulated | Tumor size | <5 vs ≥5 cm | 0.301 | 16 | 11 | 17 | 10 | [2] |
| Gao | 2020 | circ_0001721 | up-regulated | Tumor size | ≤2 vs >2 cm | 0.002 | 19 | 11 | 6 | 20 | [3] |
| Huang | 2018 | circNASP | up-regulated | Tumor size | <5 vs ≥5 cm | 0.026 | 13 | 6 | 6 | 14 | [5] |
| Jiang | 2021 | circ_0000658 | down-regulated | Tumor size | <5 vs ≥5 cm | 0.0292 | 15 | 15 | 24 | 6 | [8] |
| Jin | 2019A | circ_0102049 | up-regulated | Tumor size | <6 vs ≥6 cm | 0.022 | 16 | 22 | 6 | 32 | [9] |
| Jin | 2019B | circ_100876 | up-regulated | Tumor size | <5 vs ≥5 cm | 0.004 | 16 | 8 | 6 | 18 | [10] |
| Jin | 2019C | circ_0002052 | down-regulated | Tumor size | ≤5 vs >5 cm | 0.134 | 11 | 12 | 16 | 7 | [11] |
| Lei | 2020 | circ_0003074 | up-regulated | Tumor size | <8 vs ≥8 cm | 0.0003 | 27 | 5 | 11 | 17 | [12] |
| Li | 2018 | circ_0007534 | up-regulated | Tumor size | <6 vs ≥6 cm | 0.017 | 13 | 18 | 3 | 23 | [13] |
| Li | 2019 | circ_0001721 | up-regulated | Tumor size | <6 vs ≥6 cm | 0.046 | 9 | 19 | 2 | 22 | [14] |
| Li | 2020B | circ 0003732 | up-regulated | Tumor size | <6 vs ≥6 cm | 0.012 | 11 | 12 | 3 | 20 | [16] |
| Li | 2020C | circ_0000190 | down-regulated | Tumor size | <5 vs ≥5 cm | 0.0326 | 23 | 7 | 14 | 16 | [17] |
| Liu | 2020 | circ_100284 | up-regulated | Tumor size | ≤6 vs >6 cm | 0.0247 | 18 | 8 | 10 | 16 | [18] |
| Liu | 2021A | circ_0105346 | up-regulated | Tumor size | <8 vs ≥8 cm | 0.027 | 14 | 6 | 7 | 13 | [19] |
| Liu | 2021B | circMTO1 | down-regulated | Tumor size | ≤5 vs >5 cm | 0.858 | 17 | 21 | 15 | 17 | [20] |
| Nie | 2018 | circNT5C2 | up-regulated | Tumor size | <8 vs ≥8 cm | 0.207 | 41 | 45 | 32 | 52 | [23] |
| Pan | 2019 | circMMP9 | up-regulated | Tumor size | <5 vs ≥5 cm | 0.0229 | 18 | 6 | 11 | 16 | [24] |
| Pan | 2020 | circ_103801 | up-regulated | Tumor size | ≤5 vs >5 cm | 0.0180 | 13 | 12 | 3 | 15 | [25] |
| Qi | 2018 | circ_0000502 | up-regulated | Tumor size | <6 vs ≥6 cm | 0.006 | 16 | 18 | 4 | 25 | [26] |
| Wang | 2019B | circ_0002052 | down-regulated | Tumor size | ≤5 vs >5 cm | 0.33 | 13 | 14 | 20 | 13 | [28] |
| Wang | 2020A | circCNST | up-regulated | Tumor size | <5 vs ≥5 cm | 0.018 | 22 | 0 | 82 | 22 | [30] |
| Xiang | 2020 | circ_0005721 | up-regulated | Tumor size | <8 vs ≥8 cm | 0.500 | 11 | 14 | 10 | 15 | [36] |
| Yan | 2020 | circPVT1 | up-regulated | Tumor size | <5 vs ≥5 cm | 0.149 | 14 | 10 | 13 | 11 | [37] |
| Yang | 2020 | circ_0001105 | up-regulated | Tumor size | ≤7.5 vs >7.5 cm | 0.001 | 13 | 44 | 34 | 29 | [38] |
| Zhang | 2018 | circ_001569 | up-regulated | Tumor size | <5 vs ≥5 cm | 0.221 | 14 | 6 | 8 | 8 | [40] |
| Zhang | 2020A | circ_0002052 | up-regulated | Tumor size | <5 vs ≥5 cm | 0.0187 | 17 | 3 | 9 | 11 | [42] |
| Zhang | 2020C | circ_0017247 | up-regulated | Tumor size | ≤8 vs >8 cm | 0.737 | 9 | 13 | 11 | 13 | [44] |
| Zhang | 2021 | circ_0005909 | up-regulated | Tumor size | <8 vs ≥8 cm | 0.705 | 10 | 5 | 9 | 6 | [45] |
| Zhu | 2019 | circ_0000885 | up-regulated | Tumor size | <8 vs ≥8 cm | 0.500 | 11 | 14 | 10 | 15 | [52] |
| Chen | 2021 | circ_0000885 | up-regulated | Clinical stage | WHO I-II vs III | 0.032 | 3 | 16 | 3 | 8 | [1] |
| Ding | 2020 | circ_0005909 | up-regulated | Clinical stage | UD I-II vs III | 0.029 | 17 | 10 | 9 | 8 | [2] |
| Hu | 2020 | circLARP4 | down-regulated | Clinical stage | Enneking IIA vs IIB | 0.022 | 34 | 2 | 27 | 9 | [4] |
| Huang | 2018 | circNASP | up-regulated | Clinical stage | Enneking I-IIA vs IIB-III | 0.025 | 14 | 5 | 7 | 13 | [5] |
| Ji | 2020 | circ_001621 | up-regulated | Clinical stage | WHO I-II vs III | 0.004 | 5 | 15 | 2 | 8 | [6] |
| Jiang | 2021 | circ_0000658 | down-regulated | Clinical stage | UD I-IIA vs IIB-III | 0.0084 | 13 | 17 | 23 | 7 | [8] |
| Jin | 2019A | circ_0102049 | up-regulated | Clinical stage | WHO I-II vs III | 0.192 | 13 | 25 | 7 | 31 | [9] |
| Jin | 2019B | circ_100876 | up-regulated | Clinical stage | WHO I-II vs III-IV | 0.558 | 9 | 15 | 11 | 13 | [10] |
| Jin | 2019C | circ_0002052 | down-regulated | Clinical stage | WHO I-II vs III-IV | 0.017 | 17 | 6 | 9 | 14 | [11] |
| Lei | 2020 | circ_0003074 | up-regulated | Clinical stage | Enneking I-IIA vs IIB-III | 0.0009 | 25 | 7 | 10 | 18 | [12] |
| Li | 2018 | circ_0007534 | up-regulated | Clinical stage | WHO I-II vs III | 0.132 | 11 | 20 | 4 | 22 | [13] |
| Li | 2019 | circ_0001721 | up-regulated | Clinical stage | WHO I-II vs III | 0.023 | 15 | 13 | 5 | 19 | [14] |
| Li | 2020B | circ 0003732 | up-regulated | Clinical stage | Enneking I-IIA vs IIB-III | 0.045 | 8 | 14 | 3 | 20 | [16] |
| Li | 2020C | circ_0000190 | down-regulated | Clinical stage | UD I-IIA vs IIB-III | 0.0195 | 18 | 12 | 9 | 21 | [17] |
| Liu | 2020 | circ_100284 | up-regulated | Clinical stage | WHO I-II vs III-IV | 0.0125 | 17 | 9 | 8 | 18 | [18] |
| Liu | 2021A | circ_0105346 | up-regulated | Clinical stage | Enneking I-II vs III | 0.018 | 17 | 3 | 10 | 10 | [19] |
| Liu | 2021B | circMTO1 | down-regulated | Clinical stage | Enneking I-II vs III | 0.041 | 8 | 30 | 7 | 25 | [20] |
| Ma | 2018 | circHIPK3 | down-regulated | Clinical stage | Enneking I-IIA vs IIB-III | 0.042 | 35 | 10 | 21 | 16 | [21] |
| Nie | 2018 | circNT5C2 | up-regulated | Clinical stage | UD IIA vs IIB-III | 0.006 | 50 | 36 | 31 | 53 | [23] |
| Pan | 2019 | circMMP9 | up-regulated | Clinical stage | WHO I-II vs III-IV | 0.0047 | 18 | 6 | 9 | 18 | [24] |
| Pan | 2020 | circ_103801 | up-regulated | Clinical stage | WHO I-II vs III-IV | 0.0246 | 17 | 8 | 6 | 12 | [25] |
| Qi | 2018 | circ_0000502 | up-regulated | Clinical stage | WHO I-II vs III | 0.042 | 17 | 17 | 7 | 22 | [26] |
| Wang | 2019B | circ_0002052 | down-regulated | Clinical stage | WHO I-II vs III-IV | 0.00003 | 23 | 4 | 12 | 21 | [28] |
| Wang | 2020C | circ_0001658 | up-regulated | Clinical stage | Enneking IIA vs IIB | 0.5196 | 18 | 3 | 14 | 4 | [32] |
| Xiang | 2020 | circ_0005721 | up-regulated | Clinical stage | Enneking I-IIA vs IIB-III | 0.006 | 22 | 3 | 13 | 12 | [36] |
| Yan | 2020 | circPVT1 | up-regulated | Clinical stage | UD I-IIA vs IIB-III | 0.008 | 19 | 5 | 10 | 14 | [37] |
| Yang | 2020 | circ_0001105 | up-regulated | Clinical stage | WHO I-II vs III | 0.045 | 9 | 48 | 21 | 42 | [38] |
| Zhang | 2018 | circ_001569 | up-regulated | Clinical stage | WHO I-IIA vs IIB-III | 0.016 | 16 | 4 | 6 | 10 | [40] |
| Zhang | 2020A | circ_0002052 | up-regulated | Clinical stage | UD I-IIA vs IIB-III | 0.0104 | 5 | 15 | 4 | 6 | [42] |
| Zhang | 2020C | circ_0017247 | up-regulated | Clinical stage | UD IB-IIA vs IIB-III | 0.008 | 15 | 7 | 7 | 17 | [44] |
| Zhang | 2021 | circ_0005909 | up-regulated | Clinical stage | Enneking I-IIA vs IIB-III | 0.028 | 10 | 5 | 4 | 11 | [45] |
| Zhu | 2018A | circPVT1 | up-regulated | Clinical stage | Enneking I-IIA vs IIB-III | 0.044 | 27 | 3 | 30 | 20 | [49] |
| Zhu | 2018B | circ_0081001 | up-regulated | Clinical stage | Enneking I-IIA vs IIB-III | 0.046 | 24 | 3 | 35 | 20 | [50] |
| Zhu | 2018C | circ_0004674 | up-regulated | Clinical stage | Enneking I-IIA vs IIB-III | 0.013 | 21 | 2 | 23 | 14 | [51] |
| Zhu | 2019 | circ_0000885 | up-regulated | Clinical stage | Enneking I-IIA vs IIB-III | 0.006 | 22 | 3 | 13 | 12 | [52] |
| Hu | 2020 | circLARP4 | down-regulated | Histology | Conventional vs Others | 0.755 | 3 | 33 | 3 | 33 | [4] |
| Wang | 2020B | circTCF25 | up-regulated | Histology | Conventional vs Others | 0.875 | 3 | 23 | 3 | 21 | [31] |
| Wang | 2020C | circ_0001658 | up-regulated | Histology | Conventional vs Others | 0.416 | 2 | 19 | 4 | 14 | [32] |
| Jiang | 2021 | circ_0000658 | down-regulated | Histology | Single component vs Mixed | 0.7606 | 17 | 13 | 20 | 10 | [8] |
| Li | 2020C | circ_0000190 | down-regulated | Histology | Single component vs Mixed | 0.8609 | 9 | 21 | 8 | 22 | [17] |
| Yan | 2020 | circPVT1 | up-regulated | Histology | Single component vs Mixed | 0.849 | 6 | 18 | 5 | 19 | [37] |
| Yang | 2020 | circ_0001105 | up-regulated | Histology | Single component vs Mixed | 0.419 | 7 | 58 | 5 | 50 | [38] |
| Jin | 2019A | circ_0102049 | up-regulated | Differentiation | Well vs Poor | 0.100 | 19 | 19 | 11 | 27 | [9] |
| Jin | 2019B | circ_100876 | up-regulated | Differentiation | Well vs Poor | 0.00024 | 14 | 10 | 2 | 22 | [10] |
| Jin | 2019C | circ_0002052 | down-regulated | Differentiation | Well vs Poor | 0.139 | 10 | 13 | 15 | 8 | [11] |
| Lei | 2020 | circ_0003074 | up-regulated | Differentiation | Well vs Poor | 0.073 | 10 | 21 | 14 | 15 | [12] |
| Li | 2018 | circ_0007534 | up-regulated | Differentiation | Well vs Poor | 0.033 | 16 | 15 | 6 | 20 | [13] |
| Li | 2019 | circ_0001721 | up-regulated | Differentiation | Well vs Poor | 0.391 | 12 | 16 | 7 | 17 | [14] |
| Li | 2020B | circ 0003732 | up-regulated | Differentiation | Well vs Poor | 0.275 | 11 | 12 | 8 | 15 | [16] |
| Liu | 2021A | circ_0105346 | up-regulated | Differentiation | Well vs Poor | 0.525 | 8 | 12 | 10 | 10 | [19] |
| Liu | 2021B | circMTO1 | down-regulated | Differentiation | Well vs Poor | 0.337 | 17 | 21 | 18 | 14 | [20] |
| Qi | 2018 | circ_0000502 | up-regulated | Differentiation | Well vs Poor | 0.792 | 11 | 23 | 11 | 18 | [26] |
| Wang | 2019B | circ_0002052 | down-regulated | Differentiation | Well vs Poor | 0.40 | 13 | 14 | 19 | 14 | [28] |
| Wang | 2020B | circTCF25 | up-regulated | Differentiation | Well vs Poor | 0.029 | 19 | 7 | 8 | 16 | [31] |
| Wang | 2020C | circ_0001658 | up-regulated | Differentiation | III vs IV | 0.4934 | 17 | 4 | 16 | 2 | [32] |
| Zhang | 2021 | circ_0005909 | up-regulated | Differentiation | G1-2 vs G3-4 | 0.025 | 12 | 3 | 6 | 9 | [45] |
| Ding | 2020 | circ_0005909 | up-regulated | Metastasis | Absent vs Present | 0.014 | 17 | 10 | 8 | 19 | [2] |
| Gao | 2020 | circ_0001721 | up-regulated | Metastasis | Absent vs Present | 0.005 | 14 | 16 | 23 | 5 | [3] |
| Huang | 2018 | circNASP | up-regulated | Metastasis | Absent vs Present | 0.009 | 15 | 4 | 7 | 13 | [5] |
| Jiang | 2021 | circ_0000658 | down-regulated | Metastasis | Absent vs Present | 0.0040 | 12 | 18 | 23 | 7 | [8] |
| Jin | 2019A | circ_0102049 | up-regulated | Metastasis | Absent vs Present | 0.026 | 13 | 25 | 4 | 34 | [9] |
| Jin | 2019B | circ_100876 | up-regulated | Metastasis | Absent vs Present | 0.558 | 9 | 15 | 11 | 13 | [10] |
| Jin | 2019C | circ_0002052 | down-regulated | Metastasis | Absent vs Present | 0.006 | 19 | 4 | 10 | 13 | [11] |
| Lei | 2020 | circ_0003074 | up-regulated | Metastasis | Absent vs Present | 0.0010 | 9 | 25 | 18 | 8 | [12] |
| Li | 2018 | circ_0007534 | up-regulated | Metastasis | Absent vs Present | 0.516 | 8 | 23 | 4 | 22 | [13] |
| Li | 2019 | circ_0001721 | up-regulated | Metastasis | Absent vs Present | 0.058 | 11 | 17 | 3 | 21 | [14] |
| Li | 2020B | circ 0003732 | up-regulated | Metastasis | Absent vs Present | 0.083 | 8 | 15 | 3 | 20 | [16] |
| Li | 2020C | circ_0000190 | down-regulated | Metastasis | Absent vs Present | 0.0019 | 22 | 8 | 10 | 20 | [17] |
| Liu | 2020 | circ_100284 | up-regulated | Metastasis | Absent vs Present | 0.0110 | 20 | 6 | 11 | 15 | [18] |
| Liu | 2021A | circ_0105346 | up-regulated | Metastasis | Absent vs Present | 0.027 | 13 | 7 | 6 | 14 | [19] |
| Ma | 2018 | circHIPK3 | down-regulated | Metastasis | Absent vs Present | 0.036 | 21 | 24 | 5 | 32 | [21] |
| Nie | 2018 | circNT5C2 | up-regulated | Metastasis | Absent vs Present | 0.001 | 44 | 42 | 22 | 62 | [23] |
| Pan | 2019 | circMMP9 | up-regulated | Metastasis | Absent vs Present | 0.0246 | 17 | 7 | 10 | 17 | [24] |
| Pan | 2020 | circ_103801 | up-regulated | Metastasis | Absent vs Present | 0.6401 | 8 | 17 | 7 | 11 | [25] |
| Qi | 2018 | circ_0000502 | up-regulated | Metastasis | Absent vs Present | 0.564 | 10 | 24 | 6 | 23 | [26] |
| Wang | 2019B | circ_0002052 | down-regulated | Metastasis | Absent vs Present | 0.00019 | 24 | 3 | 14 | 19 | [28] |
| Wang | 2020A | circCNST | up-regulated | Metastasis | Absent vs Present | 1.000 | 4 | 18 | 21 | 83 | [30] |
| Wang | 2020B | circTCF25 | up-regulated | Metastasis | Absent vs Present | 0.046 | 16 | 10 | 8 | 16 | [31] |
| Xiang | 2020 | circ_0005721 | up-regulated | Metastasis | Absent vs Present | 0.009 | 10 | 15 | 2 | 23 | [36] |
| Yan | 2020 | circPVT1 | up-regulated | Metastasis | Absent vs Present | 0.009 | 18 | 6 | 9 | 15 | [37] |
| Yang | 2020 | circ_0001105 | up-regulated | Metastasis | Absent vs Present | 0.001 | 16 | 41 | 38 | 25 | [38] |
| Zhang | 2018 | circ_001569 | up-regulated | Metastasis | Absent vs Present | 0.009 | 15 | 5 | 5 | 11 | [40] |
| Zhang | 2020A | circ_0002052 | up-regulated | Metastasis | Absent vs Present | 0.0248 | 7 | 13 | 15 | 5 | [42] |
| Zhang | 2020C | circ_0017247 | up-regulated | Metastasis | Absent vs Present | 0.016 | 11 | 11 | 4 | 20 | [44] |
| Zhang | 2021 | circ_0005909 | up-regulated | Metastasis | Absent vs Present | 0.025 | 9 | 6 | 3 | 12 | [45] |
| Zhu | 2018A | circPVT1 | up-regulated | Metastasis | Absent vs Present | 0.038 | 21 | 9 | 4 | 46 | [49] |
| Zhu | 2018B | circ_0081001 | up-regulated | Metastasis | Absent vs Present | 0.024 | 21 | 6 | 4 | 51 | [50] |
| Zhu | 2019 | circ_0000885 | up-regulated | Metastasis | Absent vs Present | 0.009 | 10 | 15 | 2 | 23 | [52] |
| Hu | 2020 | circLARP4 | down-regulated | Chemotherapy | Good vs Poor | 0.032 | 11 | 25 | 16 | 20 | [4] |
| Yang | 2020 | circ_0001105 | up-regulated | Chemotherapy | Good vs Poor | 0.026 | 13 | 27 | 26 | 20 | [38] |
| Lei | 2020 | circ_0003074 | up-regulated | Chemotherapy | Not resistant vs Resistant | 0.005 | 22 | 10 | 9 | 19 | [12] |
| Zhu | 2018A | circPVT1 | up-regulated | Chemotherapy | Not resistant vs Resistant | 0.025 | 22 | 8 | 10 | 40 | [49] |
| Zhu | 2018B | circ_0081001 | up-regulated | Chemotherapy | Not resistant vs Resistant | 0.012 | 22 | 5 | 10 | 45 | [50] |
| Zhu | 2018C | circ_0004674 | up-regulated | Chemotherapy | Not resistant vs Resistant | 0.00001 | 16 | 7 | 8 | 29 | [51] |
| Lei | 2020 | circ_0003074 | up-regulated | ALP | Normal vs Abnormal | 0.041 | 20 | 9 | 13 | 18 | [12] |
| Nie | 2018 | circNT5C2 | up-regulated | ALP | Normal vs Abnormal | 0.546 | 38 | 48 | 41 | 43 | [23] |
| Zhu | 2019 | circ_0000885 | up-regulated | ALP | Normal vs Abnormal | 0.284 | 13 | 16 | 12 | 9 | [52] |

Note: E+o+ stands for patients with exposure and positive outcome, E+O- stands for patients with exposure and negative outcome, E-O+ stands for patients without exposure and positive outcome, and E-O- stands for patients without exposure and negative outcome, respectively.

Abbreviations: ALP = alkaline phosphatase, UD = undefined, WHO = World Health Organization, y/o = year old.

**Supplementary Table 4 Original data of included studies on prognosis**

| Author | Year | CircRNA | Regulation pattern | Sample size | Cutoff | Data availability | NOS | Outcome | HR | Lower 95%CI | Upper 95%CI | P value | Ref |
| --- | --- | --- | --- | --- | --- | --- | --- | --- | --- | --- | --- | --- | --- |
| Ding | 2020 | circ_0005909 | up-regulated | 54 | median | K-M curve | 5 | OS | 2.05 | 0.74 | 5.69 | **<0.01** | [2] |
| Gao | 2020 | circ_0001721 | up-regulated | 56 | median | K-M curve (p) | 4 | OS | 3.86 | 1.78 | 8.33 | **0.0006** | [3] |
| Hu | 2020 | circLARP4 | down-regulated | 72 | median | K-M curve (p) | 6 | OS | 2.28 | 1.17 | 4.41 | **0.015** | [4] |
| Ji | 2020 | circ_001621 | up-regulated | 30 | NR | K-M curve | 6 | OS | 2.56 | 1.03 | 6.35 | **0.0422** | [6] |
| Jiang | 2020 | circXPO1 | up-regulated | 52 | median | K-M curve (p) | 6 | OS | 2.45 | 1.29 | 4.65 | **0.006** | [7] |
| Jiang | 2021 | circ_0000658 | down-regulated | 60 | median | K-M curve (p) | 5 | OS | 2.23 | 1.12 | 4.44 | **0.023** | [8] |
| Jin | 2019A | circ_0102049 | up-regulated | 76 | median | Reported (HR) | 4 | OS | **1.929** | **1.903** | **3.402** | **0.023** | [9] |
| Jin | 2019B | circ_100876 | up-regulated | 48 | median | K-M curve (p) | 5 | OS | 2.51 | 1.36 | 4.63 | **0.0031** | [10] |
| Jin | 2019C | circ_0002052 | down-regulated | 46 | median | Reported (HR) | 5 | OS | **3.298** | **1.531** | **5.515** | **0.006** | [11] |
| Lei | 2020 | circ_0003074 | up-regulated | 60 | median | K-M curve (p) | 6 | OS | 2.17 | 1.08 | 4.36 | **0.029** | [12] |
| Li | 2018 | circ_0007534 | up-regulated | 57 | average | Reported (HR) | 6 | OS | **2.046** | **1.058** | **3.956** | **0.033** | [13] |
| Li | 2019 | circ_0001721 | up-regulated | 52 | average | Reported (HR) | 6 | OS | **1.928** | **1.012** | **3.763** | **0.046** | [14] |
| Li | 2020B | circ 0003732 | up-regulated | 46 | median | K-M curve | 5 | OS | 1.76 | 0.96 | 3.22 | / | [16] |
| Liu | 2020 | circ_100284 | up-regulated | 52 | median | K-M curve (HR) | 4 | OS | **2.31** | 1.15 | 4.64 | **0.0378** | [18] |
| Liu | 2021A | circ_0105346 | up-regulated | 40 | median | K-M curve (p) | 6 | OS | 2.86 | 1.35 | 6.06 | **0.006** | [19] |
| Liu | 2021B | circMTO1 | down-regulated | 70 | NR | K-M curve | 5 | OS | 1.52 | 0.93 | 2.49 | / | [20] |
| Ma | 2018 | circHIPK3 | down-regulated | 82 | median | K-M curve | 6 | OS | 1.82 | 0.91 | 3.65 | **<0.05** | [21] |
| Nie | 2018 | circNT5C2 | up-regulated | 170 | median | Reported (HR) | 7 | OS | **2.133** | **1.037** | **4.037** | **0.011** | [23] |
| Pan | 2019 | circMMP9 | up-regulated | 51 | NR | K-M curve | 4 | OS | 2.31 | 1.02 | 5.24 | **<0.05** | [24] |
| Pan | 2020 | circ_103801 | up-regulated | 43 | NR | K-M curve (p) | 3 | OS | 2.11 | 1.04 | 4.32 | **0.0397** | [25] |
| Qi | 2018 | circ_0000502 | up-regulated | 63 | median | Reported (HR) | 6 | OS | **2.292** | **1.245** | **4.220** | **0.008** | [26] |
| Wang | 2019B | circ_0002052 | down-regulated | 60 | average | Reported (HR) | 7 | OS | **3.84** | **1.64** | **9.01** | **<0.01** | [28] |
| Wang | 2020A | circCNST | up-regulated | 126 | NR | Reported (HR) | 6 | OS | **2.825** | **1.240** | **6.434** | **0.013** | [30] |
| Wei | 2021 | circ_0081001 | up-regulated | 63 | median | K-M curve (p) | 5 | OS | 3.50 | 1.23 | 9.99 | **0.019** | [33] |
| Wen | 2021 | circHIPK3 | up-regulated | 12 | NR | K-M curve (p) | 3 | OS | 5.40 | 1.74 | 16.73 | **0.0035** | [34] |
| Xiang | 2020 | circ_0005721 | up-regulated | 50 | median | K-M curve (HR) | 8 | OS | **2.458** | 1.23 | 4.90 | **0.037** | [36] |
| Yan | 2020 | circPVT1 | up-regulated | 48 | NR | K-M curve (p) | 4 | OS | 2.83 | 1.36 | 5.87 | **0.0053** | [37] |
| Yang | 2020 | circ_0001105 | up-regulated | 120 | NR | Reported (HR) | 5 | OS | **2.665** | **2.137** | **3.068** | **0.018** | [38] |
| Zhang | 2020A | circ_0002052 | up-regulated | 40 | median | K-M curve (p) | 4 | OS | 2.48 | 1.03 | 5.98 | **0.0425** | [42] |
| Zhang | 2020B | circ_0136666 | up-regulated | 47 | NR | K-M curve | 3 | OS | 2.42 | 1.07 | 5.49 | **<0.05** | [43] |
| Zheng | 2019 | circLRP6 | up-regulated | 50 | NR | Reported (HR) | 4 | OS | **2.59** | **1.75** | **3.41** | **0.017** | [47] |
| Zhou | 2017 | circ_0008717 | up-regulated | 45 | ROC | Reported (HR) | 6 | OS | **3.505** | **1.287** | **5.221** | **0.011** | [48] |
| Zhu | 2018A | circPVT1 | up-regulated | 80 | average | K-M curve (p) | 6 | OS | 2.49 | 1.40 | 4.44 | **0.002** | [49] |
| Zhu | 2018B | circ_0081001 | up-regulated | 82 | average | K-M curve (HR) | 7 | OS | **3.122** | 1.61 | 6.04 | **0.03** | [50] |
| Zhu | 2018C | circ_0004674 | up-regulated | 60 | average | K-M curve (p) | 6 | OS | 2.51 | 1.45 | 4.34 | **<0.01** | [51] |
| Zhu | 2019 | circ_0000885 | up-regulated | 50 | median | Reported (HR) | 6 | OS | **2.458** | 1.37 | 4.42 | **0.047** | [52] |
| Hu | 2020 | circLARP4 | down-regulated | 72 | median | K-M curve (p) | 6 | DFS | 2.63 | 1.43 | 4.87 | **0.002** | [4] |
| Jiang | 2020 | circXPO1 | up-regulated | 52 | median | K-M curve (p) | 6 | DFS | 2.54 | 1.37 | 4.71 | **0.003** | [7] |
| Nie | 2018 | circNT5C2 | up-regulated | 170 | median | Reported (HR) | 7 | DFS | **2.884** | **1.215** | **4.569** | **0.008** | [23] |
| Xiang | 2020 | circ_0005721 | up-regulated | 50 | median | K-M curve (HR) | 8 | DFS | **1.924** | 1.06 | 3.50 | **0.009** | [36] |
| Yang | 2020 | circ_0001105 | up-regulated | 120 | NR | Reported (HR) | 5 | DFS | **2.565** | **1.985** | **3.458** | **0.020** | [38] |
| Zheng | 2019 | circLRP6 | up-regulated | 50 | NR | Reported (HR) | 4 | DFS | **1.44** | **1.21** | **1.78** | **0.009** | [47] |
| Zhu | 2019 | circ_0000885 | up-regulated | 50 | median | K-M curve (HR) | 6 | DFS | **1.924** | 0.91 | 3.00 | **0.099** | [52] |

Note: All the data directly extracted from articles were marked in bold.

Abbreviations: CI = confidence interval, DFS = disease-free survival, HR = hazard ratio, K-M curve = Kaplan-Meier curve, NA = not applicable, NR = not reported, OS = overall survival, ROC = receiver operation curve analysis.

**Supplementary Figure 1 Forest plots of included studies on clinicopathogical features by cutoff values**

(A) Age, (B) Tumor size and (C) Clinical stage.

**Supplementary Figure 2 Forest plots of included studies on overall survival by subgroups**

(A) Regulation pattern, (B) Sample size, (C) Data availability, (D) Cut-off value and (E) Newcastle - Ottawa Scale.

**Supplementary Figure 3 Circ_0002052 and osteosarcoma**

Forest plots for the relation between circ_0002052 and (A) Age, (B) Gender, (C) Tumor site, (D) Tumor size, (E) Clinical stage, (F) Differentiation grade, (G) Metastasis, and (H) Overall survival.

**Supplementary Reference**

1. Chen Y, Zhang S, Bai C, Guan Z, Chen W. Circ_0000885 Enhances Osteosarcoma Progression by Increasing FGFR1 Expression via Sponging MiR-1294. Cancer Manag Res. 2020 Jul 28;12:6441-6452. doi: 10.2147/CMAR.S244382. PMID: 32801884; PMCID: PMC7395693.
2. Ding S, Zhang G, Gao Y, Chen S, Cao C. Circular RNA hsa_circ_0005909 modulates osteosarcoma progression via the miR-936/HMGB1 axis. Cancer Cell Int. 2020 Jul 13;20:305. doi: 10.1186/s12935-020-01399-1. PMID: 32684842; PMCID: PMC7359231.
3. Gao Y, Ma H, Gao Y, Tao K, Fu L, Ren R, Hu X, Kou M, Chen B, Shi J, Wen Y. CircRNA Circ_0001721 Promotes the Progression of Osteosarcoma Through miR-372-3p/MAPK7 Axis. Cancer Manag Res. 2020 Sep 11;12:8287-8302. doi: 10.2147/CMAR.S244527. PMID: 32982424; PMCID: PMC7498501.
4. Hu Y, Gu J, Shen H, Shao T, Li S, Wang W, Yu Z. Circular RNA LARP4 correlates with decreased Enneking stage, better histological response, and prolonged survival profiles, and it elevates chemosensitivity to cisplatin and doxorubicin via sponging microRNA-424 in osteosarcoma. J Clin Lab Anal. 2020 Feb;34(2):e23045. doi: 10.1002/jcla.23045. Epub 2019 Oct 22. PMID: 31642110; PMCID: PMC7031593.
5. Huang L, Chen M, Pan J, Yu W. Circular RNA circNASP modulates the malignant behaviors in osteosarcoma via miR-1253/FOXF1 pathway. Biochem Biophys Res Commun. 2018 Jun 2;500(2):511-517. doi: 10.1016/j.bbrc.2018.04.131. Epub 2018 Apr 21. PMID: 29678578.
6. Ji X, Shan L, Shen P, He M. Circular RNA circ_001621 promotes osteosarcoma cells proliferation and migration by sponging miR-578 and regulating VEGF expression. Cell Death Dis. 2020 Jan 6;11(1):18. doi: 10.1038/s41419-019-2204-y. PMID: 31907361; PMCID: PMC6944700.
7. Jiang Y, Hou J, Zhang X, Xu G, Wang Y, Shen L, Wu Y, Li Y, Yao L. Circ-XPO1 upregulates XPO1 expression by sponging multiple miRNAs to facilitate osteosarcoma cell progression. Exp Mol Pathol. 2020 Dec;117:104553. doi: 10.1016/j.yexmp.2020.104553. Epub 2020 Oct 19. PMID: 33091396.
8. Jiang X, Chen D. Circular RNA hsa_circ_0000658 inhibits osteosarcoma cell proliferation and migration via the miR-1227/IRF2 axis. J Cell Mol Med. 2021 Jan;25(1):510-520. doi: 10.1111/jcmm.16105. Epub 2020 Dec 2. PMID: 33264494; PMCID: PMC7810968.
9. Jin Y, Li L, Zhu T, Liu G. Circular RNA circ_0102049 promotes cell progression as ceRNA to target MDM2 via sponging miR-1304-5p in osteosarcoma. Pathol Res Pract. 2019 Dec;215(12):152688. doi: 10.1016/j.prp.2019.152688. Epub 2019 Oct 12. PMID: 31727503.
10. Jin J, Chen A, Qiu W, Chen Y, Li Q, Zhou X, Jin D. Dysregulated circRNA_100876 suppresses proliferation of osteosarcoma cancer cells by targeting microRNA-136. J Cell Biochem. 2019 Sep;120(9):15678-15687. doi: 10.1002/jcb.28837. Epub 2019 May 8. PMID: 31069828.
11. Jin Z, Cui J, Zeng Y, Zhou Q, Liu J. Expression of circular RNA circ_0002052 in osteosarcoma and its clinical significance. Journal of Chinese Practical Diagnosis and Therapy. 2019;33(1):32-34. doi: 10.13507/j.isn.1674-3474.2019.01.010. [Article in Chinese].
12. Lei S, Xiang L. Up-Regulation of circRNA hsa_circ_0003074 Expression is a Reliable Diagnostic and Prognostic Biomarker in Patients with Osteosarcoma. Cancer Manag Res. 2020 Sep 29;12:9315-9325. doi: 10.2147/CMAR.S262093. PMID: 33061621; PMCID: PMC7532912.
13. Li B, Li X. Overexpression of hsa_circ_0007534 predicts unfavorable prognosis for osteosarcoma and regulates cell growth and apoptosis by affecting AKT/GSK-3β signaling pathway. Biomed Pharmacother. 2018 Nov;107:860-866. doi: 10.1016/j.biopha.2018.08.086. Epub 2018 Aug 22. PMID: 30142548.
14. Li L, Guo L, Yin G, Yu G, Zhao Y, Pan Y. Upregulation of circular RNA circ_0001721 predicts unfavorable prognosis in osteosarcoma and facilitates cell progression via sponging miR-569 and miR-599. Biomed Pharmacother. 2019 Jan;109:226-232. doi: 10.1016/j.biopha.2018.10.072. Epub 2018 Nov 2. PMID: 30396080.
15. Li X, Liu Y, Zhang X, Shen J, Xu R, Liu Y, Yu X. Circular RNA hsa_circ_0000073 contributes to osteosarcoma cell proliferation, migration, invasion and methotrexate resistance by sponging miR-145-5p and miR-151-3p and upregulating NRAS. Aging (Albany NY). 2020 Jul 24;12(14):14157-14173. doi: 10.18632/aging.103423. Epub 2020 Jul 24. PMID: 32706759; PMCID: PMC7425447.
16. Li S, Pei Y, Wang W, Liu F, Zheng K, Zhang X. Extracellular nanovesicles-transmitted circular RNA has_circ_0000190 suppresses osteosarcoma progression. J Cell Mol Med. 2020 Feb;24(3):2202-2214. doi: 10.1111/jcmm.14877. Epub 2020 Jan 10. PMID: 31923350; PMCID: PMC7011131.
17. Li L, Kong XA, Zang M, Dong J, Feng Y, Gui B, Hu Y. Hsa_circ_0003732 promotes osteosarcoma cells proliferation via miR-545/CCNA2 axis. Biosci Rep. 2020 Jun 26;40(6):BSR20191552. doi: 10.1042/BSR20191552. PMID: 32537647; PMCID: PMC7313442.
18. Liu J, Li X, Yue L, Lv H. Circ_0105346 Knockdown Inhibits Osteosarcoma Development via Regulating miR-1182/WNT7B Axis. Cancer Manag Res. 2021 Jan 20;13:521-535. doi: 10.2147/CMAR.S281430. PMID: 33505171; PMCID: PMC7829129.
19. Liu YD, Liu LP. Circ100284 promotes invasion and migration of osteosarcoma cells by down-regulating PTEN and EMP1. Eur Rev Med Pharmacol Sci. 2020 Jun;24(12):6540-6550. doi: 10.26355/eurrev_202006_21638. PMID: 32633341.
20. Liu DY, Li Z, Zhang K, Jiao N, Lu DG, Zhou DW, Meng YB, Sun L. Circular RNA CircMTO1 suppressed proliferation and metastasis of osteosarcoma through miR-630/KLF6 axis. Eur Rev Med Pharmacol Sci. 2021 Jan;25(1):86-93. doi: 10.26355/eurrev_202101_24352. PMID: 33506896.
21. Ma XL, Zhu KP Z, Zhang CL. Circular RNA circ_HIPK3 is down-regulated and suppresses cell proliferation, migration and invasion in osteosarcoma. J Cancer. 2018 Apr 26;9(10):1856-1862. doi: 10.7150/jca.24619. PMID: 29805712; PMCID: PMC5968774.
22. Mao X, Guo S, Gao L, Li G. Circ-XPR1 promotes osteosarcoma proliferation through regulating the miR-214-5p/DDX5 axis. Hum Cell. 2021 Jan;34(1):122-131. doi: 10.1007/s13577-020-00412-z. Epub 2020 Sep 12. PMID: 32920730.
23. Nie WB, Zhao LM, Guo R, Wang MX, Ye FG. Circular RNA circ-NT5C2 acts as a potential novel biomarker for prognosis of osteosarcoma. Eur Rev Med Pharmacol Sci. 2018 Oct;22(19):6239-6244. doi: 10.26355/eurrev_201810_16030. PMID: 30338784.
24. Pan G, Hu T, Chen X, Zhang C. Upregulation Of circMMP9 Promotes Osteosarcoma Progression Via Targeting miR-1265/CHI3L1 Axis. Cancer Manag Res. 2019 Oct 29;11:9225-9231. doi: 10.2147/CMAR.S226264. PMID: 31754311; PMCID: PMC6825504.
25. Pan Y, Lin Y, Mi C. Cisplatin-resistant osteosarcoma cell-derived exosomes confer cisplatin resistance to recipient cells in an exosomal circ_103801-dependent manner. Cell Biol Int. 2020 Dec 15. doi: 10.1002/cbin.11532. Epub ahead of print. PMID: 33325136.
26. Qi H, Sun Y, Jiang Y, Li X. Upregulation of circular RNA circ_0000502 predicts unfavorable prognosis in osteosarcoma and facilitates cell progression via sponging miR-1238. J Cell Biochem. 2018 Dec 7. doi: 10.1002/jcb.28134. Epub ahead of print. PMID: 30525215.
27. Wang L, Du ZG, Huang H, Li FS, Li GS, Xu SN. Circ-0003998 promotes cell proliferative ability and invasiveness by binding to miR-197-3p in osteosarcoma. Eur Rev Med Pharmacol Sci. 2019 Dec;23(24):10638-10646. doi: 10.26355/eurrev_201912_19761. PMID: 31858530.
28. Wang C, Feng XB, Qu JH. Expression of circ-0002052 in osteosarcoma and its correlation with clinicopathological features and prognosis. Journal of Clinical and Experimental Medicine. 2019;18(23): 2521-2524. doi: 10.3969/j.issn.1671-4695.2019.23.017. [Article in Chinese].
29. Wang L, Zhang GC, Kang FB, Zhang L, Zhang YZ. hsa_circ0021347 as a Potential Target Regulated by B7-H3 in Modulating the Malignant Characteristics of Osteosarcoma. Biomed Res Int. 2019 Dec 17;2019:9301989. doi: 10.1155/2019/9301989. PMID: 31950059; PMCID: PMC6948356.
30. Wang JH, Wu XJ, Duan YZ, Li F. Circular RNA_CNST Promotes the Tumorigenesis of Osteosarcoma Cells by Sponging miR-421. Cell Transplant. 2020 Jan-Dec;29:963689720926147. doi: 10.1177/0963689720926147. PMID: 32693639; PMCID: PMC7563816.
31. Wang Y, Shi S, Zhang Q, Dong H, Zhang J. MicroRNA-206 upregulation relieves circTCF25-induced osteosarcoma cell proliferation and migration. J Cell Physiol. 2020 Feb 13. doi: 10.1002/jcp.29570. Epub ahead of print. PMID: 32052450.
32. Wang L, Wang P, Su X, Zhao B. Circ_0001658 promotes the proliferation and metastasis of osteosarcoma cells via regulating miR-382-5p/YB-1 axis. Cell Biochem Funct. 2020 Jan;38(1):77-86. doi: 10.1002/cbf.3452. Epub 2019 Nov 22. PMID: 31758574.
33. Wei W, Ji L, Duan W, Zhu J. Circular RNA circ_0081001 knockdown enhances methotrexate sensitivity in osteosarcoma cells by regulating miR-494-3p/TGM2 axis. J Orthop Surg Res. 2021 Jan 13;16(1):50. doi: 10.1186/s13018-020-02169-5. PMID: 33435987; PMCID: PMC7805151.
34. Wen Y, Li B, He M, Teng S, Sun Y, Wang G. circHIPK3 promotes proliferation and migration and invasion via regulation of miR‑637/HDAC4 signaling in osteosarcoma cells. Oncol Rep. 2021 Jan;45(1):169-179. doi: 10.3892/or.2020.7833. Epub 2020 Nov 3. PMID: 33416147; PMCID: PMC7709833.
35. Wu Z, Shi W, Jiang C. Overexpressing circular RNA hsa_circ_0002052 impairs osteosarcoma progression via inhibiting Wnt/β-catenin pathway by regulating miR-1205/APC2 axis. Biochem Biophys Res Commun. 2018 Aug 25;502(4):465-471. doi: 10.1016/j.bbrc.2018.05.184. Epub 2018 Jun 5. PMID: 29852168.
36. Xiang L, Zhang W. Expression level of circular RNA hsa_circ_0005721 in tissues and serum of patients with osteosarcoma and its clinical significance. Journal of Clinical and Pathological Research. 2020;40(5):1136-1143. doi: 10.3978/ j.issn.2095-6959.2020.05.010. [Article in Chinese].
37. Yan M, Gao H, Lv Z, Liu Y, Zhao S, Gong W, Liu W. Circular RNA PVT1 promotes metastasis via regulating of miR-526b/FOXC2 signals in OS cells. J Cell Mol Med. 2020 May;24(10):5593-5604. doi: 10.1111/jcmm.15215. Epub 2020 Apr 5. PMID: 32249539; PMCID: PMC7214167.
38. Yang J, Han Q, Li C, Yang H, Chen X, Wang X. Circular RNA circ_0001105 Inhibits Progression and Metastasis of Osteosarcoma by Sponging miR-766 and Activating YTHDF2 Expression. Onco Targets Ther. 2020 Feb 26;13:1723-1736. doi: 10.2147/OTT.S234668. PMID: 32161468; PMCID: PMC7051899.
39. Zhang H, Wang G, Ding C, Liu P, Wang R, Ding W, Tong D, Wu D, Li C, Wei Q, Zhang X, Li D, Liu P, Cui H, Tang H, Ji F. Increased circular RNA UBAP2 acts as a sponge of miR-143 to promote osteosarcoma progression. Oncotarget. 2017 Jun 27;8(37):61687-61697. doi: 10.18632/oncotarget.18671. PMID: 28977896; PMCID: PMC5617456.
40. Zhang H, Yan J, Lang X, Zhuang Y. Expression of circ_001569 is upregulated in osteosarcoma and promotes cell proliferation and cisplatin resistance by activating the Wnt/β-catenin signaling pathway. Oncol Lett. 2018 Nov;16(5):5856-5862. doi: 10.3892/ol.2018.9410. Epub 2018 Sep 6. PMID: 30344736; PMCID: PMC6176349.
41. Zhang Z, Zhao M, Wang G. Hsa_circ_0051079 functions as an oncogene by regulating miR-26a-5p/TGF-β1 in osteosarcoma. Cell Biosci. 2019 Nov 29;9:94. doi: 10.1186/s13578-019-0355-2. PMID: 31798828; PMCID: PMC6883546.
42. Zhang PR, Ren J, Wan JS, Sun R, Li Y. Circular RNA hsa_circ_0002052 promotes osteosarcoma via modulating miR-382/STX6 axis. Hum Cell. 2020 Jul;33(3):810-818. doi: 10.1007/s13577-020-00335-9. Epub 2020 Apr 9. PMID: 32274658.
43. Zhang C, Zhou H, Yuan K, Xie R, Chen C. Overexpression of hsa_circ_0136666 predicts poor prognosis and initiates osteosarcoma tumorigenesis through miR-593-3p/ZEB2 pathway. Aging (Albany NY). 2020 May 18;12(11):10488-10496. doi: 10.18632/aging.103273. Epub 2020 May 18. PMID: 32424109; PMCID: PMC7346030.
44. Zhang C, Na N, Liu L, Qiu Y. CircRNA hsa_circ_0005909 Promotes Cell Proliferation of Osteosarcoma Cells by Targeting miR-338-3p/HMGA1 Axis. Cancer Manag Res. 2021 Jan 27;13:795-803. doi: 10.2147/CMAR.S285118. PMID: 33536787; PMCID: PMC7850455.
45. Zhang G, Wang X, Zhu L. Highly expressed circular RNA hsa_circ_0017247 promotes osteosarcoma cell invasion and migration. Chinese Journal of Cellular and Molecular Immunology. 2021;36(7):634-639. doi: 10.13423/j.cnki.cjcmi.009037. [Article in Chinese].
46. Zhao Y, Zhang J. CircSAMD4A accelerates cell proliferation of osteosarcoma by sponging miR-1244 and regulating MDM2 mRNA expression. Biochem Biophys Res Commun. 2019 Aug 13;516(1):102-111. doi: 10.1016/j.bbrc.2019.05.182. Epub 2019 Jun 11. PMID: 31200957.
47. Zheng S, Qian Z, Jiang F, Ge D, Tang J, Chen H, Yang J, Yao Y, Yan J, Zhao L, Li H, Yang L. CircRNA LRP6 promotes the development of osteosarcoma *via* negatively regulating KLF2 and APC levels. Am J Transl Res. 2019 Jul 15;11(7):4126-4138. PMID: 31396323; PMCID: PMC6684910.
48. Zhou X, Natino D, Qin Z, Wang D, Tian Z, Cai X, Wang B, He X. Identification and functional characterization of circRNA-0008717 as an oncogene in osteosarcoma through sponging miR-203. Oncotarget. 2017 Dec 20;9(32):22288-22300. doi: 10.18632/oncotarget.23466. PMID: 29854278; PMCID: PMC5976464.
49. Zhu KP, Ma XL, Zhang CL. Overexpressed circPVT1, a potential new circular RNA biomarker, contributes to doxorubicin and cisplatin resistance of osteosarcoma cells by regulating ABCB1. Int J Biol Sci. 2018 Feb 12;14(3):321-330. doi: 10.7150/ijbs.24360. PMID: 29559849; PMCID: PMC5859477.
50. Zhu KP, Zhang CL, Hu JP, Zhang L. A novel circulating hsa_circ_0081001 act as a potential biomarker for diagnosis and prognosis of osteosarcoma. Int J Biol Sci. 2018 Sep 1;14(11):1513-1520. doi: 10.7150/ijbs.27523. PMID: 30263004; PMCID: PMC6158732.
51. Zhu KP, Ma XL, Zhang L, Zhang CL, Hu JP, Cai T, Zhan TC. Screening circular RNA related to chemotherapeutic resistance in osteosarcoma by RNA sequencing. Epigenomics. 2018 Oct;10(10):1327-1346. doi: 10.2217/epi-2018-0023. Epub 2018 Sep 7. PMID: 30191736.
52. Zhu K, Niu L, Wang J, Wang Y, Zhou J, Wang F, Cheng Y, Zhang Q, Li H. Circular RNA hsa_circ_0000885 Levels are Increased in Tissue and Serum Samples from Patients with Osteosarcoma. Med Sci Monit. 2019 Feb 25;25:1499-1505. doi: 10.12659/MSM.914899. PMID: 30802235; PMCID: PMC6400018.
53. Mitchell M, Muftakhidinov B, Winchen T, et al. Engauge Digitizer Software. Accessed via <http://markummitchell.github.io/engauge-digitizer> on 2021-02-10. doi: 10.5281/zenodo.3941227.
54. Tierney JF, Stewart LA, Ghersi D, Burdett S, Sydes MR. Practical methods for incorporating summary time-to-event data into meta-analysis. Trials. 2007 Jun 7;8:16. doi: 10.1186/1745-6215-8-16. PMID: 17555582; PMCID: PMC1920534.
